# Coding of odors by temporal binding within a model network of the locust antennal lobe

**DOI:** 10.3389/fncom.2013.00050

**Published:** 2013-04-25

**Authors:** Mainak J. Patel, Aaditya V. Rangan, David Cai

**Affiliations:** ^1^Department of Mathematics, Duke UniversityDurham, NC, USA; ^2^Courant Institute of Mathematical Sciences, New York UniversityNew York, NY, USA; ^3^Department of Mathematics, MOE-LSC, Institute of Natural Sciences, Shanghai Jiao Tong UniversityShanghai, China; ^4^Courant Institute of Mathematical Sciences and Center for Neural Science, New York UniversityNew York, NY, USA; ^5^NYUAD Institute, New York University Abu DhabiAbu Dhabi, UAE

**Keywords:** antennal lobe, temporal binding, computational neuroscience, odor coding, slow temporal patterns, oscillations, synchrony, time scales of inhibition

## Abstract

The locust olfactory system interfaces with the external world through antennal receptor neurons (ORNs), which represent odors in a distributed, combinatorial manner. ORN axons bundle together to form the antennal nerve, which relays sensory information centrally to the antennal lobe (AL). Within the AL, an odor generates a dynamically evolving ensemble of active cells, leading to a stimulus-specific temporal progression of neuronal spiking. This experimental observation has led to the hypothesis that an odor is encoded within the AL by a dynamically evolving trajectory of projection neuron (PN) activity that can be decoded piecewise to ascertain odor identity. In order to study information coding within the locust AL, we developed a scaled-down model of the locust AL using Hodgkin–Huxley-type neurons and biologically realistic connectivity parameters and current components. Using our model, we examined correlations in the precise timing of spikes across multiple neurons, and our results suggest an alternative to the dynamic trajectory hypothesis. We propose that the dynamical interplay of fast and slow inhibition within the locust AL induces temporally stable correlations in the spiking activity of an odor-dependent neural subset, giving rise to a temporal binding code that allows rapid stimulus detection by downstream elements.

## Introduction

**Figure 1:**
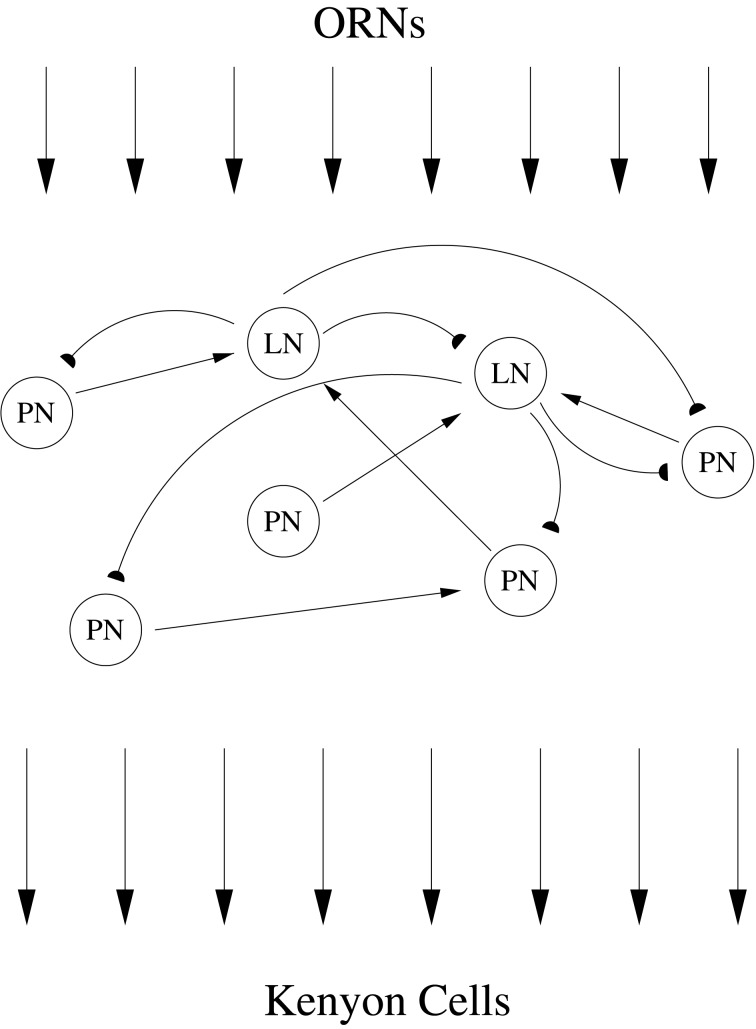
**ORNs within the locust antennae detect odors in the environment, relaying a particular pattern of receptor neuron activation to LNs and PNs within the AL.** Interacting excitatory PNs and inhibitory LNs within the AL network process and reformat odor information. PNs provide the sole output of the AL, and after exiting the AL PN axons relay processed odor information to Kenyon cells within the mushroom body.

The locust antennal lobe (AL) can be deconstructed within the framework of stimulus encoding, providing an excellent system in which to study early sensory processing. The AL consists of excitatory projection neurons (PNs) and inhibitory local neurons (LNs) which receive odor information from olfactory receptor neurons (ORNs) within the antennae that detect environmental odors. After processing of odor information within the AL, PNs relay the result to Kenyon cells (KCs) within the mushroom body (Figure 1). Examination of AL network behavior via analysis of spike trains obtained through intracellular recordings reveals a complex odor response. The responses of PNs to a stimulus show slow patterning—i.e., the firing rate of each PN exhibits a reproducible, slowly-varying temporal structure that is dependent on both PN and odor identity and can significantly outlast the stimulus (Laurent et al., 1996). As shown through local field potential (LFP) recordings, this slow patterning of PN firing rates is superimposed on a global 20 Hz network oscillation that is critically dependent on fast GABAergic transmission by LNs within the AL (Laurent and Davidowitz, 1994; Laurent and Naraghi, 1994; MacLeod and Laurent, 1996). Application of picrotoxin to the AL to block fast GABA_A_ receptors abolishes the global 20 Hz oscillation but preserves slow patterning (MacLeod and Laurent, 1996; MacLeod et al., 1998), hence eliminating synchrony while leaving firing rates undisturbed. Collectively, these features imply that the AL odor response consists of synchronized bursts of PN spikes occurring in 50 ms time steps, with the precise subset of PNs that contribute spikes to each burst evolving gradually from one oscillation cycle to the next in an odor-specific manner (Wehr and Laurent, 1996).

Decoding of a long dynamic trajectory of PN spiking requires the insect to await the unfolding of a procession of phase-locked PN subsets prior to odor identification, a lengthy process. Additionally, the insect would need to integrate this slowly evolving dynamic profile over time, which would likely entail elaborate network mechanisms downstream of the AL (within the mushroom body). In light of these factors, authors have provided experimental evidence that odor information is contained within the composition of individual PN assemblies, and hence it is not necessary to decode temporal sequences of phase-locked PN activity (i.e., that dynamic PN trajectories can be decoded piecewise) (Laurent, 2002; Stopfer et al., 2003; Brown et al., 2005; Mazor and Laurent, 2005; Broome et al., 2006). Piecewise decoding implies that individual oscillation cycles can contain enough information to pinpoint stimulus identity, but, since the composition of the phase-locked PN ensemble evolves through time from cycle to cycle, the internally stored template for an odor must depend on the oscillation cycle [although, since PN activity evolves gradually, the template for a particular oscillation cycle may be useful for temporally adjacent oscillation cycles as well—e.g., see Figure 7 of Brown et al. (2005)].

In contrast to piecewise decoding, we theorize that there exists a code hidden within the spatiotemporal patterns of PN spiking described above, a code that could take the form of higher order correlations embedded within network dynamics. Instead of an odor code that dynamically evolves through time, we postulate that an odor is encoded as a temporally bound neural subset (i.e., a stimulus-specific ensemble of PNs with the property that, whenever any one member of the ensemble fires a spike, a large proportion of the other ensemble members fire in rapid succession). Our temporal binding hypothesis asserts that there exists a subset of synchronously firing, temporally bound PNs that is stable from cycle to cycle, and that it is this stable temporally bound subset that is actually coding for the odor. It is therefore not necessary to compare PN activity to an odor template that changes with time—a single, time-invariant odor template is sufficient, and all that is required is to observe a single synchronous firing event of this stable temporally bound neural subset within any oscillation cycle to ascertain odor identity. Hence, piecewise decoding of a dynamic trajectory is a time-dependent process, while a temporal binding code provides a time-invariant way to decipher odor information.

Odor-induced temporal binding of PN activity has been observed in the moth AL, and investigators have shown that such correlated activity can carry information about stimulus attributes (Christensen et al., 2000; Lei et al., 2002). In order to investigate temporal binding within the locust, we constructed a biologically plausible network model of the locust AL, and we showed in a previous paper that by using realistic currents and parameters our model is capable of capturing experimentally observed features of locust AL physiology (Patel et al., 2009). In this work, we demonstrate that a temporal binding code does indeed emerge from the dynamics of our model, and that this code is dependent on the intricate interplay of fast and slow inhibition within the network. Within our model, we show that fast GABA_A_-type synapses, rather than merely organizing PN spikes into coherent bursts and causing brief (50–150 ms) periods of correlated activity, are also responsible for the emergence of long-term correlations in PN firing (i.e., the temporal binding code). Additionally, we show that slow inhibition within our model, which activates ~500 ms after odor onset, selectively quiets those PNs which comprise the odor-induced temporally bound subset, allowing the temporal binding code to specifically signal odor onset. Thus, the two inhibitory components within our model serve opposing functions—fast inhibition serves to generate the temporal binding code, while slow inhibition serves to silence temporally bound PNs once a newly appearing odor has been detected. Since the two inhibitory time scales play antagonistic roles, we independently examined the effects of one versus the other by carrying out simulations in which either only fast or slow inhibition is present within the model network.

## Results

Our model AL consisted of Hodgkin–Huxley-type cells (90 PNs and 30 LNs) with sparse cell-type specific (i.e., PN–PN, LN–LN, LN–PN, PN–LN) connection probabilities consistent with experiment, yielding a randomly generated but fixed wiring diagram. In accordance with experiment, PNs within our network fired fast (~3 ms) sodium spikes and formed fast excitatory cholinergic synapses (via nicotinic receptors) with other neurons, while model LNs fired slow calcium spikes (~25 ms) and formed fast inhibitory GABAergic synapses (via fast GABA_A_ receptors) with other neurons. Moreover, we endowed the network with a postulated (but not yet experimentally verified) synaptic slow inhibitory current from LNs to PNs acting over ~150–200 ms, a current possibly mediated via slow metabotropic receptors. An odor was simulated by sending stimulus current to a subset of 36 PNs and 12 LNs within the network, with different odors represented as different subsets of stimulated PNs and LNs (see Methods for details).

In a previous paper (Patel et al., 2009), we showed that our model exhibits (a) GABA-dependent 20 Hz LFP oscillations that decay over the first second of stimulation, (b) slow temporal patterning of PN responses generated by the slow inhibitory current from LNs to PNs, and (c) preservation of slow patterning after removal of fast GABA synapses to abolish the network oscillation. In this work, we will extend these results to show that, when the precise timing of PN spikes is examined, a temporal binding code is seen to emerge from the dynamics of our model. We begin by showing that when network activity is examined over a long time window (1 s), correlated PN firing occurs only when two conditions are satisfied: (1) fast GABAergic inhibition is present within the network; (2) slow inhibition is absent from the network. This suggests that fast GABAergic inhibition is necessary to induce temporal correlations in PN activity, while slow inhibition suppresses PNs firing in a correlated fashion. We then show that the correlated PN firing induced by GABA in the absence of slow inhibition actually does exist in networks with both fast and slow inhibition, as long as one looks over a short enough time window (~500 ms) following odor onset. Thus, we find that in networks with both fast and slow inhibition, fast GABA synapses induce temporally correlated PN activity shortly following odor onset, while slow inhibitory synapses activate ~500 ms after odor onset and selectively suppress PNs firing in a correlated fashion. Furthermore, we show that (in a network with both fast and slow inhibition) temporally correlated PN activity, while present, can be used to construct odor-specific subsets of temporally bound PNs that allow hypothetical KCs to rapidly and accurately classify odors. To assess the effects of GABA dynamics, we performed simulations in which the strength of fast GABA synapses was doubled or tripled; other than an increase in magnitude of the 20 Hz peak in the LFP power spectrum, we note that firing rates and network oscillations are left unscathed by these modifications, and hence this entire range of GABA conductances is consistent with plausible network models of the locust AL.

### Different networks

In our investigation of the mechanisms underlying the network dynamics of our model AL, we conducted a variety of numerical experiments. The numerical experiments which illustrate our hypotheses most clearly involve comparing the dynamics between different model AL networks with varying biophysical features (e.g., different synaptic coupling strengths governing GABA_A_-type inhibition), but with the same connectivity/wiring diagram (i.e., equivalent architecture). We will later discuss the behavior of seven different networks which operate in different dynamical regimes:

- (I) fully intact network with GABA and slow inhibitory synapses as in Patel et al. (2009);
- (NG) the network with GABA strength set to 0 and normal slow inhibition;
- (2X GABA) fully intact network with doubled GABA strength;
- (3X GABA) fully intact network with tripled GABA strength;
- (NS) the network with slow inhibition strength set to 0 and normal GABA strength;
- (NS, 2X GABA) network with no slow inhibition and doubled GABA strength;
- (NS, 3X GABA) network with no slow inhibition and tripled GABA strength.

In each of these networks, the connectivity diagram associated with each neuron (of a given index) is the same. Thus, we can selectively compare the behavior of a given PN in the intact network (I) with the behavior of the “same” (i.e., equivalently wired) PN in the dynamic regime produced in the absence of slow-inhibitory conductance (the NS network), or with the “same” PN in any other network with modified synaptic strengths.

### Correlated triplets

Since our goal was to determine if a temporal binding code emerged from the dynamics of our model, we needed to detect precise temporal correlations in spiking activity among multiple PNs within our network. While standard measures exist for detecting correlated activity between two neurons, there exist no such standard measures for assessing correlations in spiking within a group consisting of more than two neurons. We therefore devised our own measure [the synchrony ratio (SR)] for detecting correlations in the activity of PN triplets. We chose to examine correlated firing within triplets of PNs for two reasons: (1) this allows the detection of correlated activity among multiple (more than two) PNs; (2) examining all possible PN triplets is computationally tractable (the combinatorics of examining PNs in groups of larger than three lead to prohibitively long simulation times).

**Figure 2:**
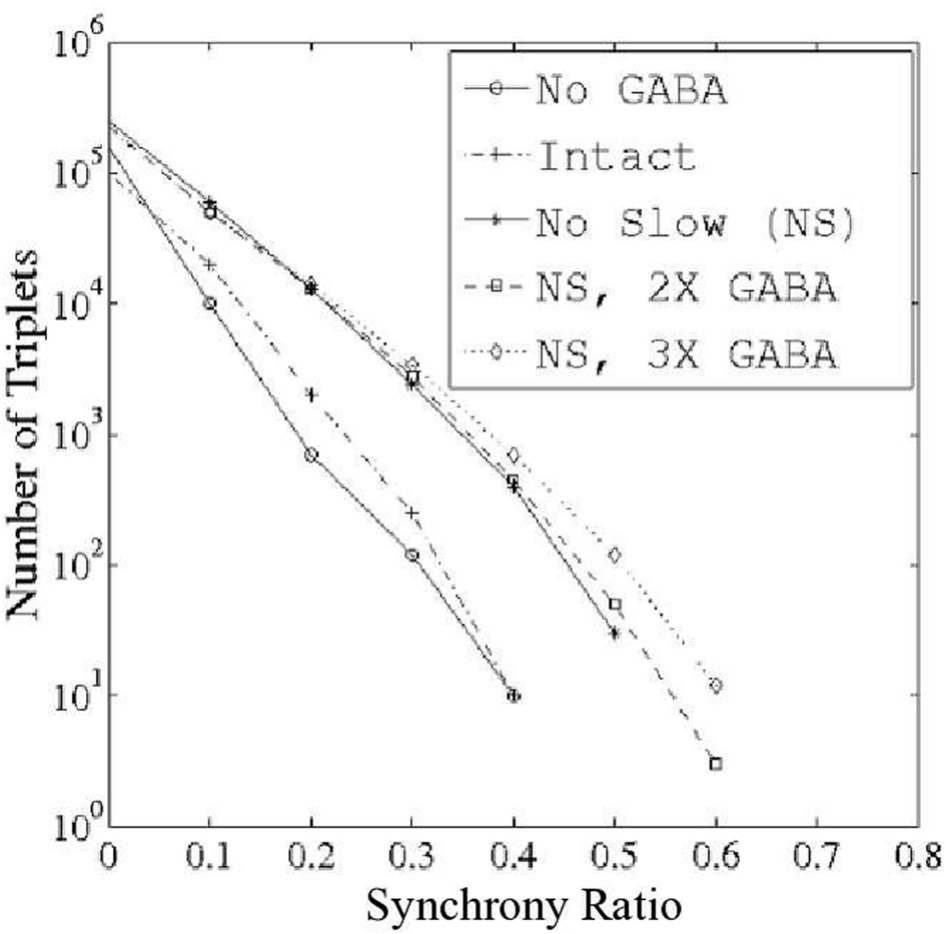
**Number of correlated PN triplets found at varying threshold values of the synchrony ratio (see Methods).** Triplets were sought during 1 s of stimulus presentation over 80 trials in networks with varying functional states: (1) network with no GABA (NG); (2) intact network (I); (3) network with no slow inhibition (NS); (4) network with no slow inhibition and double GABA (NS, 2X GABA); and (5) network with no slow inhibition and triple GABA (NS, 3X GABA).

We examined PNs in groups of three in an effort to determine the existence of triplets that fired synchronously more often than would be expected from the individual PN spike rates, or from paired neuronal correlations. To quantify this phenomenon, we devised a measure on the space of ordered PN triplets that we termed the synchrony ratio (SR). The SR for a triplet *i,j,k* of PNs was computed as described in the Methods; for the purposes of this discussion, it is sufficient to note that the SR takes values in the interval [−1,1], where a value close to zero implies that PNs *i,j,k* fire independently and values approaching unity imply that PNs *i,j,k* exhibit highly correlated firing (values approaching −1, which we do not examine, would imply negative correlations—i.e., that PNs *i,j,k* exhibit a tendency to not fire together). We emphasize that SR values approaching unity capture synchronous triplet firing which is a consequence of bona-fide 3-point correlations; the SR for a given triplet remains close to zero if the three neurons fire often together simply due to high firing rates, or due to high 2-point correlations (the latter situation can occur if a firing event of one neuron causes the other two neurons to fire independently, but with high probability). Figure 2 shows the number of triplets found at progressively greater threshold values of the SR for varying functional states of the network: network with no GABA (NG), intact network (I), network with no slow inhibition (NS), network with NS and doubled GABA strength (NS, 2X GABA), network with NS and tripled GABA strength (NS, 3X GABA). The networks with functioning slow inhibition and doubled or tripled GABA strength (2X GABA; 3X GABA), which are not plotted, behave similar to the I. While correlated triplets disappear from the I and NG networks once we impose thresholds greater than SR = 0.4, networks lacking slow inhibition but with active GABA conductances contain correlated triplets up to a threshold of SR = 0.6. Additionally, when slow inhibitory synapses are severed the number of correlated triplets found at a given SR threshold increases with the strength of GABA synapses. These results suggest that slow inhibition tends to disrupt correlated firing while GABA induces temporal correlations among PNs. Importantly, the data shown in Figure 2 represent integration over a large time-window (1 s after odor onset), which is sufficiently large to allow for slow inhibition to take effect within the I. As we will see later, the dynamics of the I produced shortly after odor onset (within ~500 ms) are more similar to the dynamics produced by the NS network.

### Temporal binding of triplets

**Figure 3:**
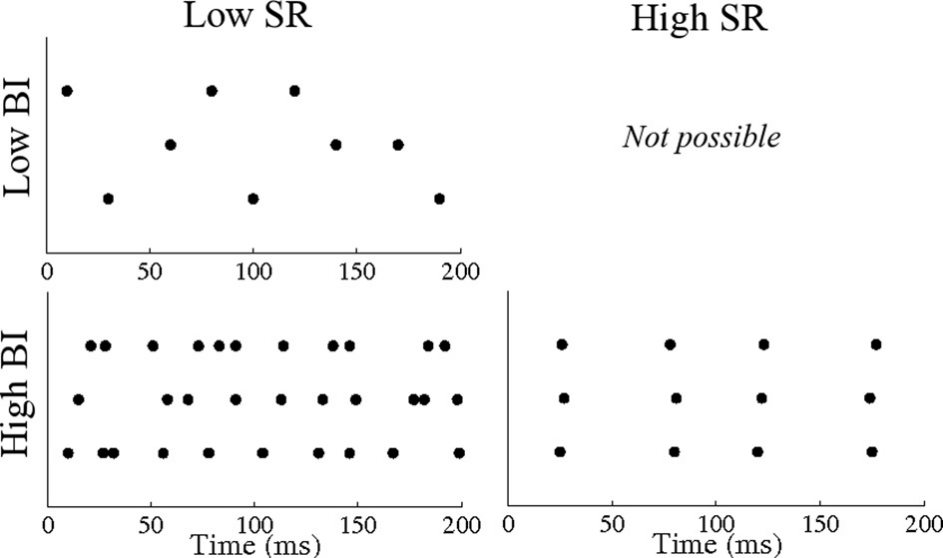
**Sample PN triplet spike rasters illustrating the difference between the synchrony ratio (SR) and binding index (BI) measures.** A triplet of PNs that fire in an uncorrelated fashion with low rate have a low SR and a low BI. A triplet of PNs that fire in an uncorrelated fashion with high rate have a low SR but a high BI. A triplet of PNs that fire in a correlated fashion with low rate have a high SR and a high BI.

To quantify temporal binding more directly, we constructed a measure on the space of unordered PN triplets taking values in the interval [0,1] that we termed the binding index (BI); for a triplet *i,j,k* of PNs, BI_*i*, *j*, *k*_ = *b* implies that, whenever any one of the PNs *i, j*, or *k* fires, the other two PNs will fire concurrently with at least a probability *b* (thus, BI_*i*, *j*, *k*_ = 0 implies that PNs *i,j,k* never fire together, while BI_*i*, *j*, *k*_ = 1 implies that PNs *i,j,k* always fire synchronously; see Methods for details). Importantly, the binding index is high for a given triplet if single firing events of each individual member of that triplet tend to be temporally adjacent to firing events of the other two members of that triplet. Thus, the BI of a given triplet can be high even if the neurons comprising that triplet fire independently (but with high rate), or possess strong 2-point correlations without having strong 3-point correlations. Notably, even though the BI for such triplets would be high (close to 1), the SR for such triplets would be low (close to 0). Figure 3 shows triplet spike rasters that illustrate the difference between the SR and BI measures. Using the SR, we have shown that temporal correlations across PNs emerge in the presence of GABAergic inhibition but are suppressed in the presence of the slow inhibitory current. However, within the locust, decoders of PN activity detect synchrony, regardless of whether synchrony is a consequence of correlated activity or high firing rates. We therefore use the BI, a direct measure of synchrony, to examine PN triplets within our model rather than the SR.

**Figure 4:**
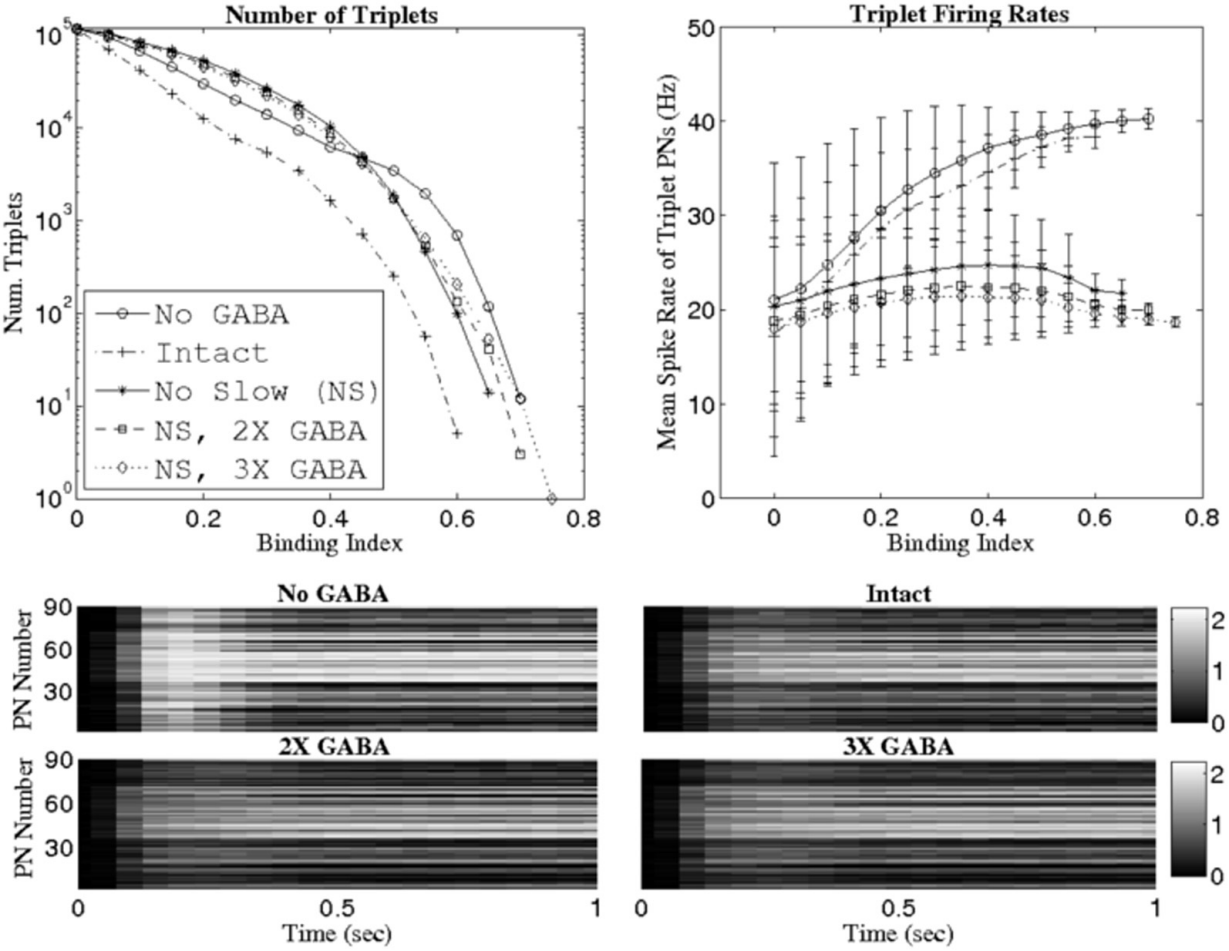
**Number of PN triplets found (**top row, left panel**) and mean firing rate of PNs comprising those triplets (**top row, right panel**) at various threshold values of the binding index (see Methods).** Binding indices are computed using data from 1 s of stimulus presentation over 80 trials. The **bottom panels** show mean PN firing rates during 1 s of stimulus presentation (averaging is performed over 80 trials) in the network with no GABA (NG), the intact network (I), the intact network with doubled GABA strength (2X GABA), and the intact network with tripled GABA strength (3X GABA). The scale in the gray-scale bars refers to spikes per 50 ms time bin.

Figure 4 plots the number of triplets found at various threshold values of the binding index (top row, left panel) as well as the mean firing rate of the PNs comprising these triplets (top row, right panel). Similar to the SR results, removal of slow inhibition (NS; NS, 2X GABA; NS, 3X GABA) leads to the persistence of triplets even at relatively high BI thresholds, while in the intact case (I) no such triplets emerge from network dynamics. As with the SR, the networks with functioning slow inhibition and doubled or tripled GABA strength (2X GABA; 3X GABA), which are not plotted, behave similar to the I. However, unlike in the case of the SR, triplets are now found at high BI thresholds after inactivation of GABA receptors (NG network); since the SR measures correlated firing while the binding index measures temporal binding, this suggests that the presence of coherent triplets without the influence of GABA synapses may be a consequence of high firing rates rather than true 3-point temporal correlations across PNs. This hypothesis is supported by the observation that as the BI threshold is increased, the mean firing rate of PNs comprising the corresponding triplets increases in the NG network and remains low in the networks devoid of slow inhibition but with substantial GABA conductances (we also note that the variance in the mean PN firing rate is close to zero in all networks for neurons that comprise triplets possessing a high BI). Additionally, even though the slow temporal structure of PN responses is unaltered by changes in the strength of GABA synapses, complete removal of GABAergic transmission tends to elevate stimulus-evoked PN firing rates in general (Figure 4, lower panels).

**Figure 5:**
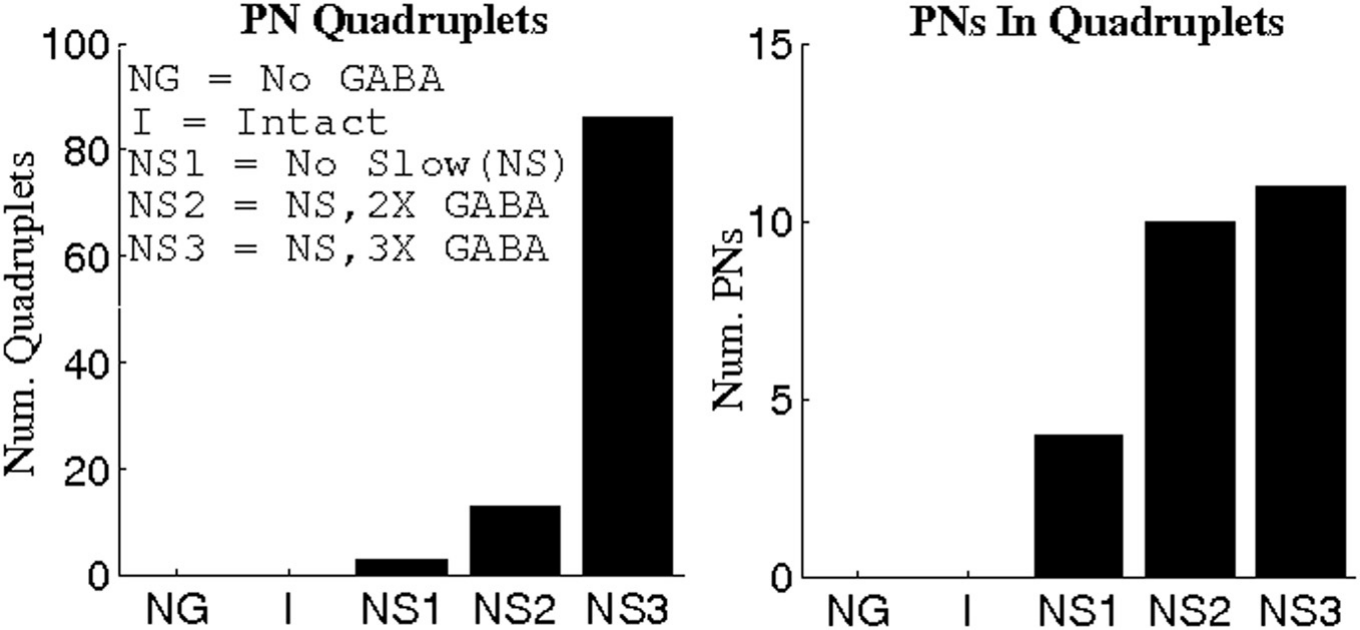
**Number of PN quadruplets found (left panel) and the number of PNs comprising these quadruplets (right panel) at a threshold value of 0.55 for the binding index (see Methods).** Binding indices are computed using data from 1 s of stimulus presentation over 80 trials.

To obtain further evidence that severing GABA synapses leads to rate-induced PN triplets rather than 3-point correlated firing, we computed binding indices for quadruplets of PNs. As shown in Figure 5, a threshold of BI = 0.55 yields quadruplets of synchronous PNs only when significant GABA-mediated currents are present and unhindered by slow inhibition; in particular, the triplets that were found in the absence of GABAergic transmission did not give rise to temporally bound quadruplets. This suggests that without GABA synapses, temporal binding does not occur and the apparent PN coherence is an artifact of high firing rates, while GABA-induced synchrony is more likely a manifestation of correlated activity across multiple PNs.

### Role of slow inhibition

**Figure 6:**
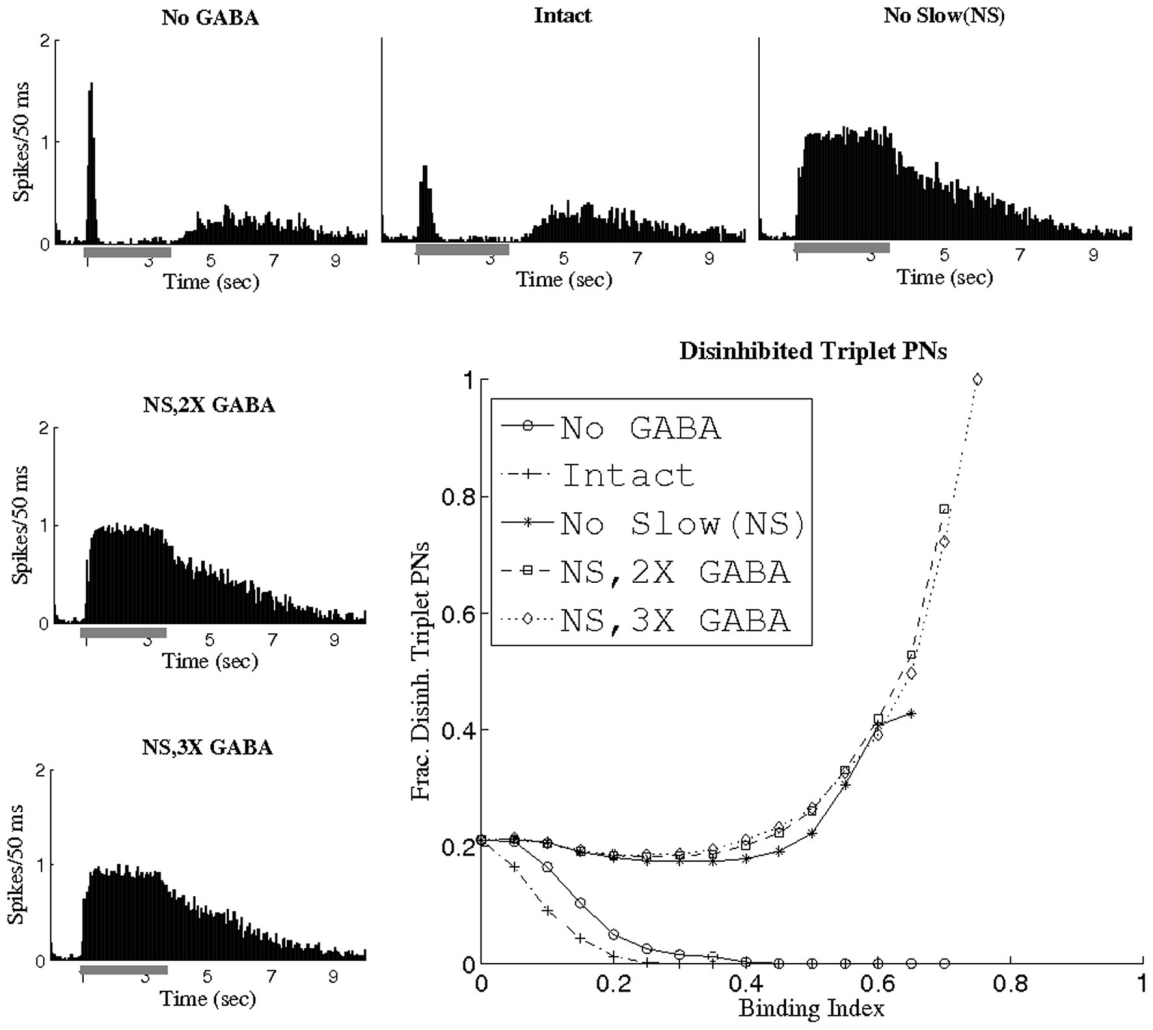
**Fraction of triplet PNs classified as disinhibited plotted as a function of the binding index threshold used to extract triplets.** The histograms show the firing rate (averaged over 80 trials, the scale refers to spikes per 50 ms bin) of a sample disinhibited PN (gray bar represents the stimulus). A disinhibited PN was defined to be a cell whose time-averaged firing rate at least doubled after removal of slow inhibition.

Since the slow inhibitory current appears to thwart the ability of GABA to generate temporal correlations, we investigated the relationship between cells receiving considerable slow inhibitory input in the I and the cells comprising correlated triplets when slow conductances were abolished (NS; NS, 2X GABA; NS, 3X GABA networks). Because the distribution of slow inhibitory synapses from LNs to PNs was identical to that of fast GABA synapses, presynaptic LN depolarization resulted in the activation of both slow and fast postsynaptic receptors, so that each PN received proportional amounts of slow and fast inhibitory input. We would therefore expect that if PNs showing a high degree of temporal coherence in the absence of slow inhibition were those that received the most GABA input, then in the presence of slow synapses these PNs would also receive substantial slow inhibitory input, and hence would tend to exhibit significantly reduced firing rates. In order to examine this phenomenon, we defined a disinhibited PN as a cell whose odor-evoked firing rate (averaged over 1 s) at least doubled following elimination of slow inhibitory synapses (i.e., PN *k* was classified as disinhibited if the firing rate of PN *k* within the NS network was at least double the firing rate of PN *k* within the I network). Thus, PNs which are tagged as disinhibited are those that receive high levels of slow inhibition in the I (and hence exhibit low firing rates in the I but show ample spiking activity in the NS network). Figure 6 plots the fraction of triplet PNs classified as disinhibited as a function of the BI threshold used to extract triplets, as well as showing spike rate histograms from a sample disinhibited PN. At a threshold of BI = 0 (i.e., when all possible PN triplets are considered), the fraction of disinhibited PNs is 0.2, indicating that 20% of PNs in the entire network were classified as disinhibited. As the BI threshold is increased, the fraction of disinhibited PNs rises steeply in the networks lacking slow inhibition but with active GABA synapses while it quickly drops to zero in the I and NG networks (since in these cases slow inhibition remains active and disinhibited PNs scarcely fire). Furthermore, non-functional slow receptors lead to triplets persisting at progressively higher BI thresholds as GABA synapses are strengthened; in fact, a three-fold amplification of maximal GABA conductances yields triplets at a threshold of BI = 0.75 that consist entirely of disinhibited PNs. These results imply that in the I, the slow inhibitory current specifically suppresses the activity of those PNs which would have exhibited temporally correlated firing in its absence. These results are consistent with the modeling work of Bazhenov et al. (2001a), in which increased presynaptic LN activity was shown to be associated with periods of decreased PN spiking in the presence of a slow inhibitory current from LNs to PNs.

### Odor specificity of triplets

**Figure 7:**
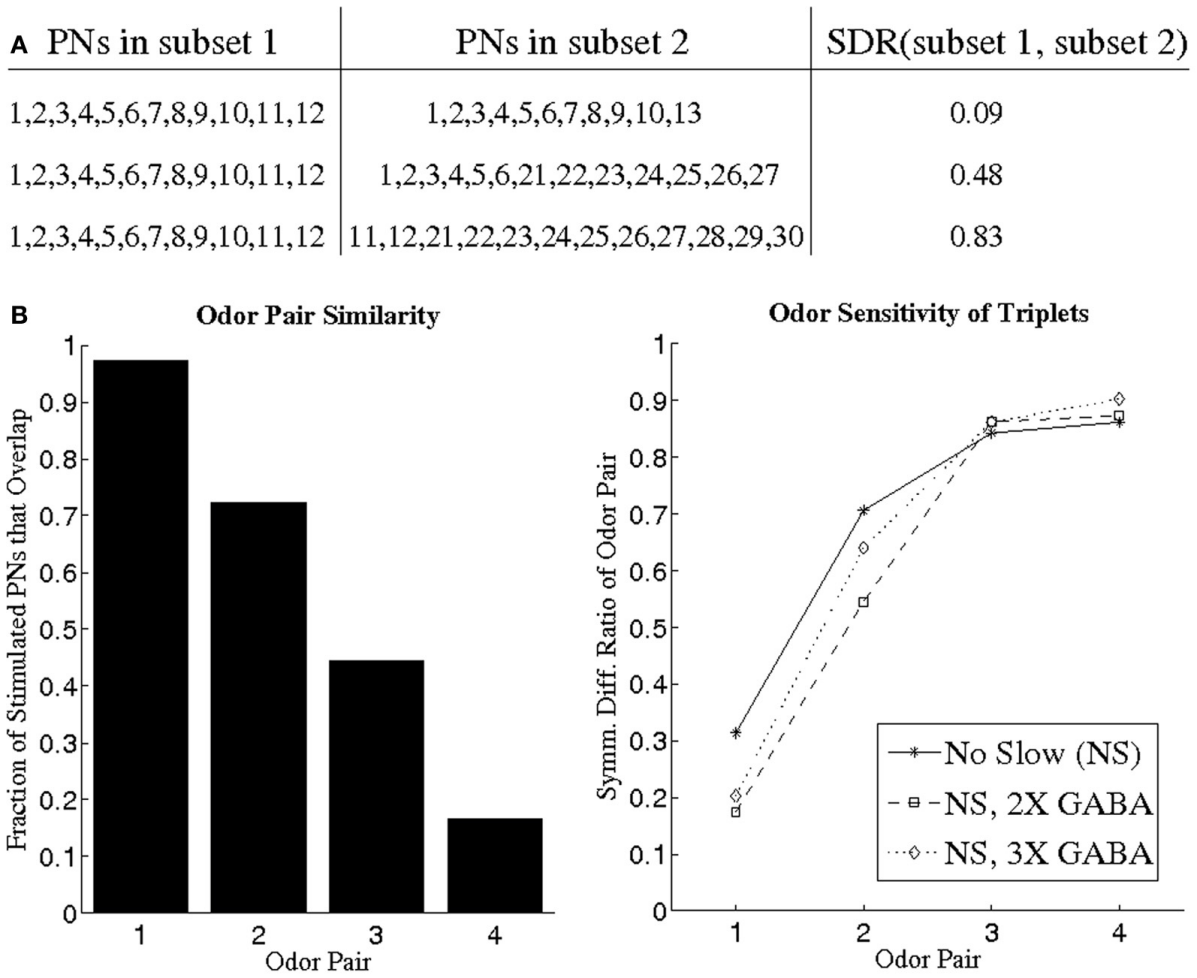
**(A)** Illustration of the symmetric difference ratio (SDR) by application to several sample pairs of PN subsets. Two PN subsets comprised of nearly identical PNs have a SDR close to 0, while the SDR of two PN subsets rises and approaches 1 as the two subsets overlap less and less. The two PN subsets need not have identical cardinality in order to apply the SDR measure (see Methods for details). **(B)** We devised four different pairs of odors (left panel), with odor pair 1 consisting of two very similar odors and odor pair 4 consisting of two very dissimilar odors (similarity between two odors is measured as the fraction of stimulated PNs that overlap between the two odors). For a given odor pair, we imposed a BI threshold of 0.65 to obtain PN triplets for each odor (the PN triplets for one odor in a pair consisted of a total of ~12 PNs). The ~12 PNs obtained for a particular odor in a pair were designated as the PN subset representative of that odor. For each of the four odor pairs, we plot the SDR of the two PN subsets representative of each odor in the pair (right panel).

While it is certainly interesting that GABA is capable of inducing temporal coherence among triplets of PNs, it is important to verify that these triplets occur in an odor-specific fashion, rather than occurring solely as a consequence of network architecture, in order for them to be utilized as a neural coding tool. We simulated different odors by stimulating differing subsets of network neurons, and we examined the stimulus dependence of synchronous triplets using pairs of odors. For a given odor pair, we employed a BI threshold of 0.65 to extract temporally bound triplets for each odor from networks devoid of slow inhibition but with operational GABA currents (since correlated PN activity is only found in the NS; NS, 2X GABA; NS, 3X GABA networks). For a given odor pair (say odor 1 and odor 2), the triplets found for each odor in any given network consisted of a total of ~12 PNs. For each network, the ~12 PNs corresponding to odor 1 were designated as the temporally bound subset representative of odor 1, and the ~12 PNs corresponding to odor 2 were designated as the temporally bound subset representative of odor 2. In order to compare the composition of the temporally bound neural subsets corresponding to odor 1 and odor 2, we designed the symmetric difference ratio (SDR) as a normalized quantity that measures the overlap between two subsets of PNs. The SDR takes values close to 0 for two subsets of PNs that very similar in composition, while SDR values approaching 1 imply nearly disjoint PN subsets (Figure 7A; see Methods for details). Figure 7B plots the SDR for pairs of odors exhibiting a progressively greater degree of divergence in the set of stimulated PNs; as the odors within a pair become more dissimilar, the SDR rises and approaches unity in all three networks examined. It is crucial to note that even for pairs of similar odors the SDR assumes non-zero values, indicating that temporally bound PN triplets are odor-dependent and sensitive to small variations in the input. If we carry out a similar analysis in networks with slow inhibition, but look only over the initial portion of the odor response to detect triplets, then similar results to those presented in Figure 7B are obtained.

### The binding code

Thus far, we have shown the existence of temporally bound PN triplets only when the dynamical effects of fast GABA synapses are unopposed by the influence of a slow inhibitory current. However, to serve as a physically reasonable mechanism by which the locust AL could potentially encode information, temporal binding of PN triplets must occur within a biologically realistic version of our model network (i.e., in the presence of both a fast GABA conductance that generates global oscillations as well as a slower conductance capable of modulating neural firing rates over prolonged time scales). The seemingly incongruous nature of these observations prompted an examination of the rise time constant of slow inhibition in our network, and we noticed that following stimulus onset the slow inhibitory current required several hundred milliseconds to grow in strength and reach a steady-state. This led us to the possibility that a GABA-dependent binding code was indeed embedded in the dynamics of the I, but that such a code was undetected by our methodology because it materialized only within a small time window close to stimulus onset before temporally bound PNs were selectively silenced by slow inhibition. We therefore propose that there exist stimulus-sensitive, temporally bound subsets of neurons within the AL that specifically signal odor onset; moreover, we hypothesize that fast inhibition is responsible for the emergence of this temporal binding code, while the function of slow inhibition is to quiet temporally bound neurons once a newly encountered odor has been detected by the animal.

**Figure 8:**
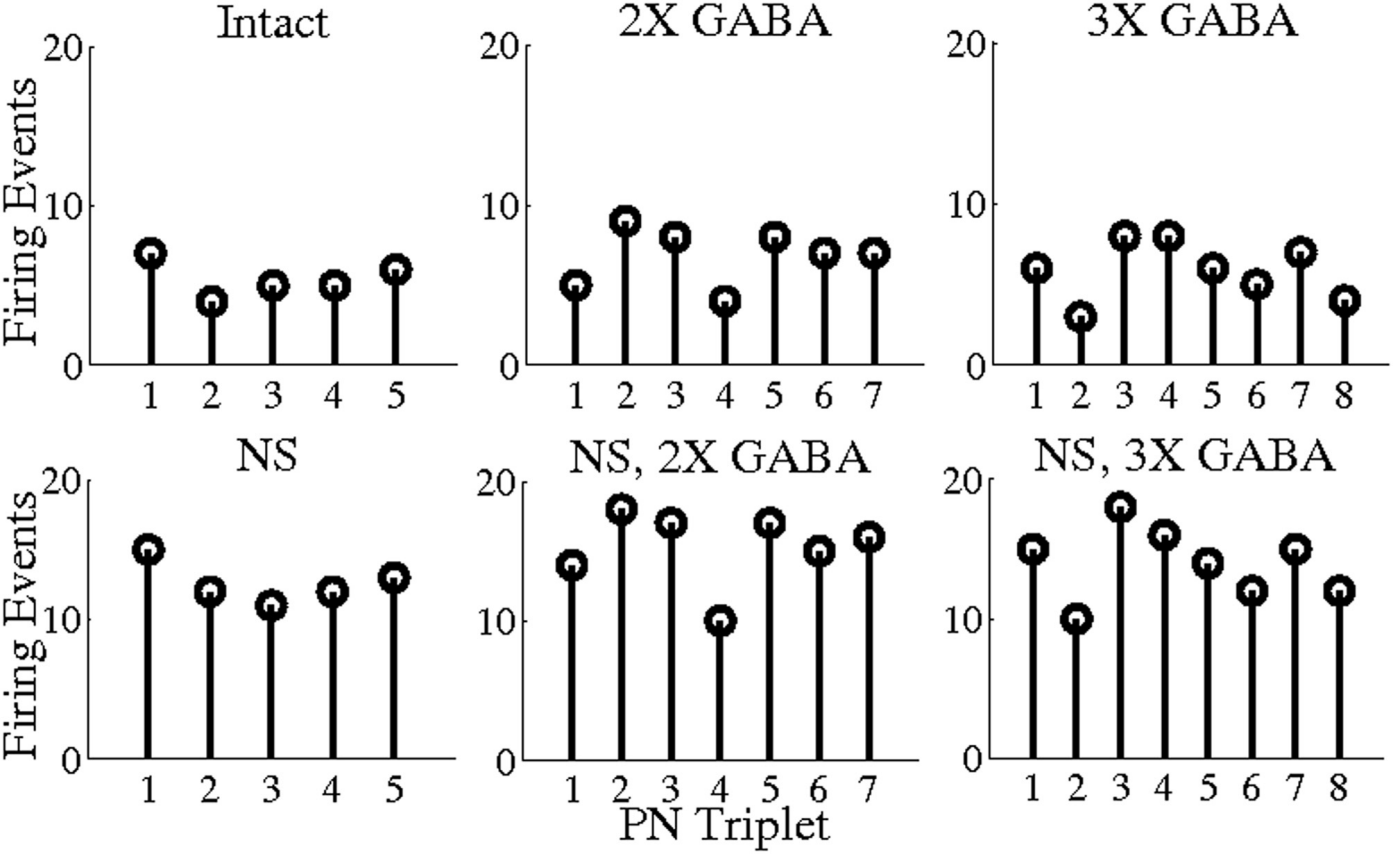
**Number of PN triplet firing events during a single 1 s odor trial.** Triplets were extracted from the networks with no slow inhibition (NS; NS, 2X GABA; NS, 3X GABA) using a threshold of BI = 0.65 (5, 7, and 8 triplets were obtained from the NS; NS, 2X GABA; NS, 3X GABA networks, respectively). The number of firing events of each triplet during a representative 1 s odor trial is plotted both within the network with no slow inhibition and in the corresponding network with slow inhibition.

**Figure 9:**
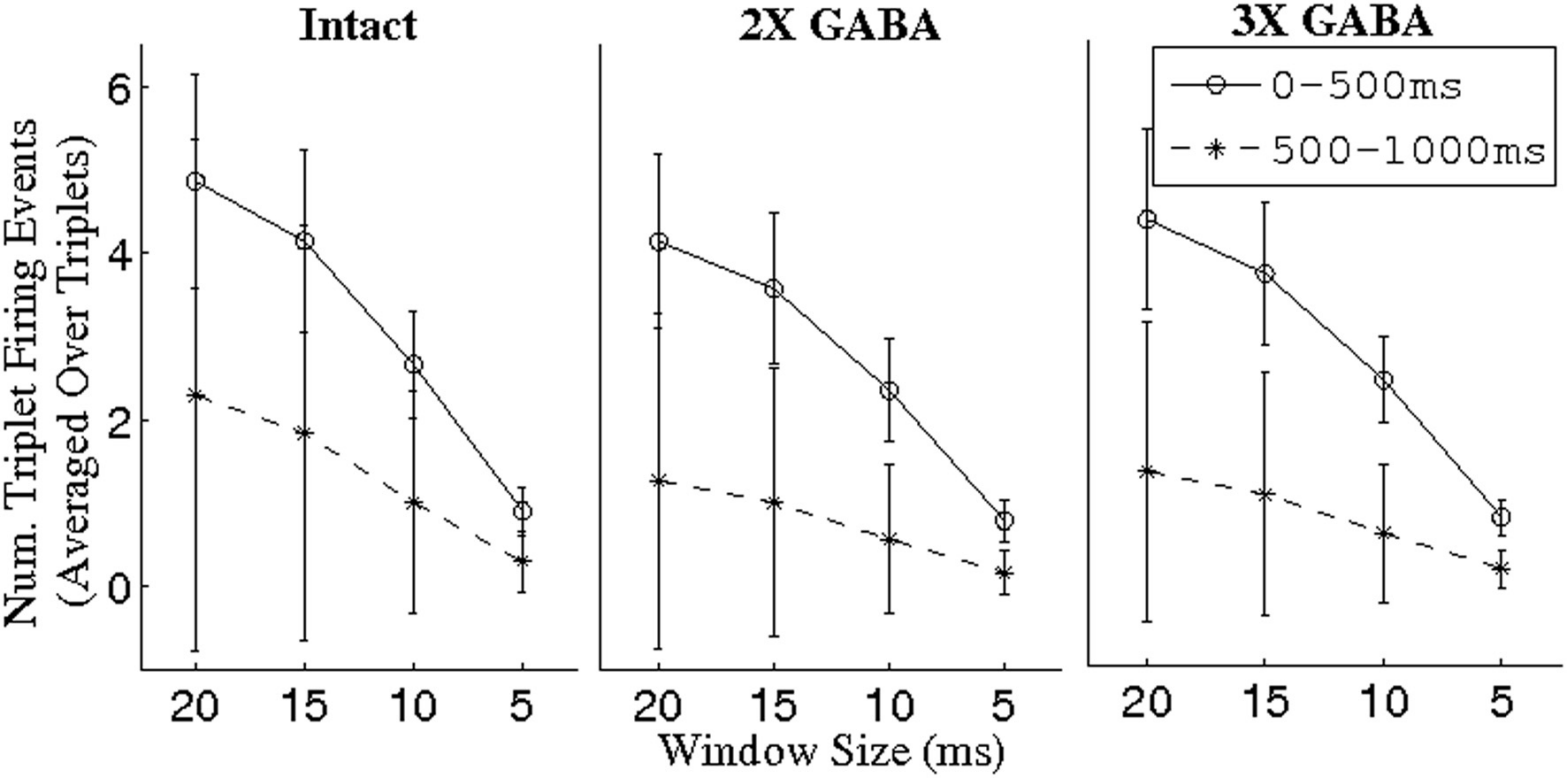
**Number of PN triplet firing events during the first and second 500 ms of the odor response in intact networks with varying maximal GABA conductances (I; 2X GABA; 3X GABA), plotted as a function of the time window in which a triplet was constrained to fire to have given rise to a synchronous event.** Triplets were extracted from the corresponding networks with no slow inhibition (NS; NS, 2X GABA; NS, 3X GABA) using a threshold of BI = 0.65. For a given triplet, firing events were counted over 80 trials and divided by 80 to obtain the number of events per trial; the number of triplet firing events per trial was subsequently averaged over triplets (the standard deviation refers to the latter mean).

**Figure 10:**
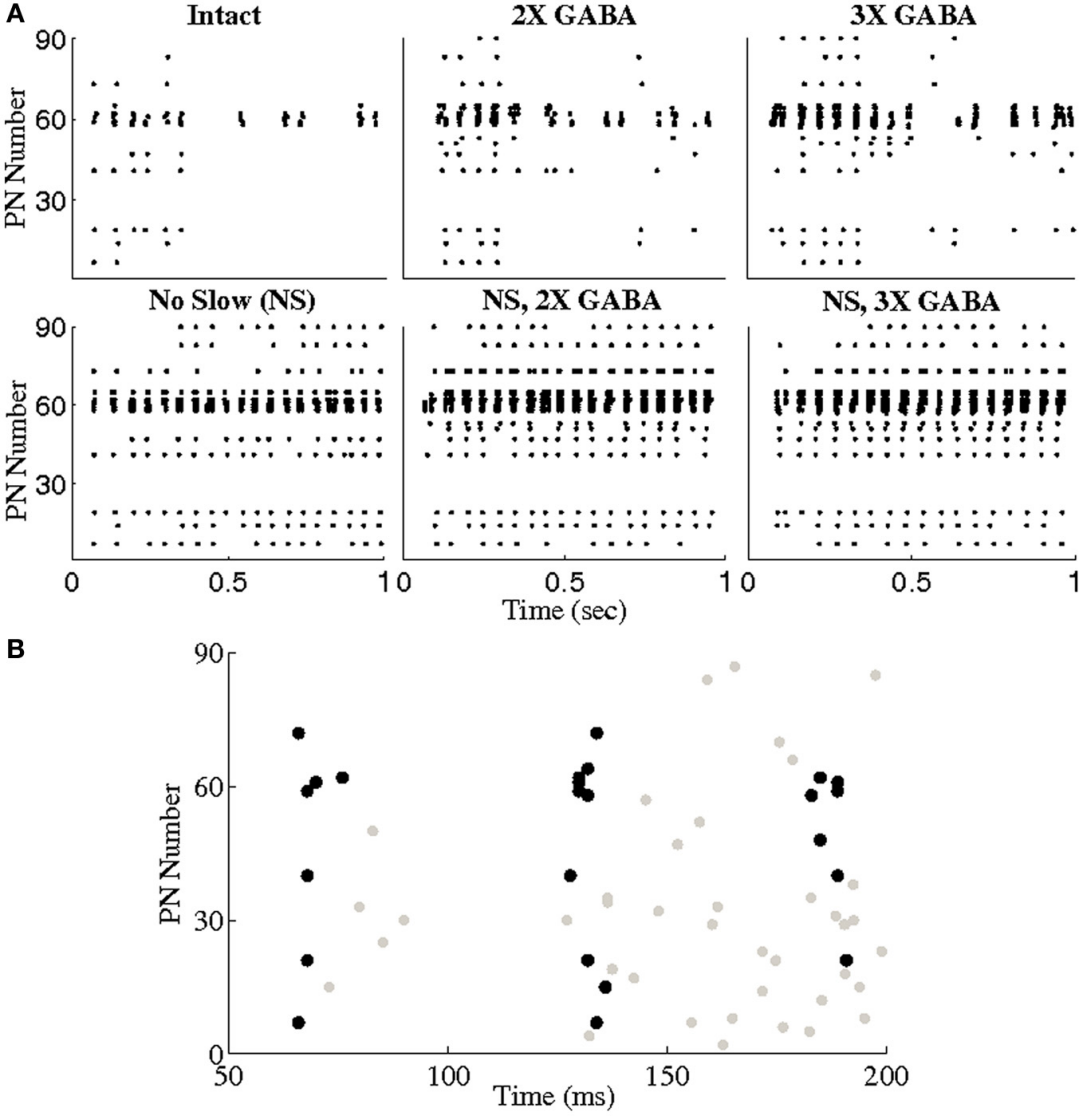
**(A)** Spike rasters from a representative trial in which only spikes from triplet firing events are plotted (a triplet of PNs was required to fire within a 10 ms window to be considered a synchronous event). Triplets were extracted from the networks with no slow inhibition (NS; NS, 2X GABA, NS, 3X GABA) using a threshold of BI = 0.65. Triplet firing events in both the networks with no slow inhibition and the corresponding intact networks (I; 2X GABA; 3X GABA) are shown. **(B)** Zoom-in of the first few oscillation cycles in the intact network. Spikes from non-triplet PNs are shown in gray.

To explore this hypothesis, we used a threshold of BI = 0.65 to extract triplets from networks lacking slow conductances, and we studied the incidence of synchronous triplet firing events in the corresponding networks with intact slow receptors. Figure 8 depicts the number of firing events of each extracted triplet during a representative 1 s odor trial in both the networks without slow inhibition and in the corresponding networks with slow inhibition, showing that temporally bound triplets actually do fire in the presence of slow inhibition, though firing events are fewer in number than in the case that slow inhibition is abolished. To assess whether this discrepancy is a consequence of triplets firing at odor onset and subsequently ceasing to fire in the networks with slow inhibition, we plot in Figure 9 the average number of triplet firing events during the first 500 ms of the odor response versus during the second 500 ms of the odor response in the networks with functional slow inhibition. Figure 9 shows that, regardless of the width of the time window used to calculate the BI, triplets tend to fire more often during the first 500 ms of the odor response than during the second 500 ms. Figure 10 shows spike rasters from an odor trial in which only spikes from synchronous triplet firing events are plotted (time window = 10 ms); while triplet firing events persisted at a high frequency throughout the odor response in the absence of slow inhibition, triplets displayed sparse firing after the first 500 ms of the odor response when slow synapses remained functional. Furthermore, the spike rasters appear to show synchrony across triplets (i.e., firing events across triplets tend to occur within similar temporal windows), suggesting that the PNs comprising these triplets may form odor-specific, temporally bound subsets of neurons.

**Figure 11:**
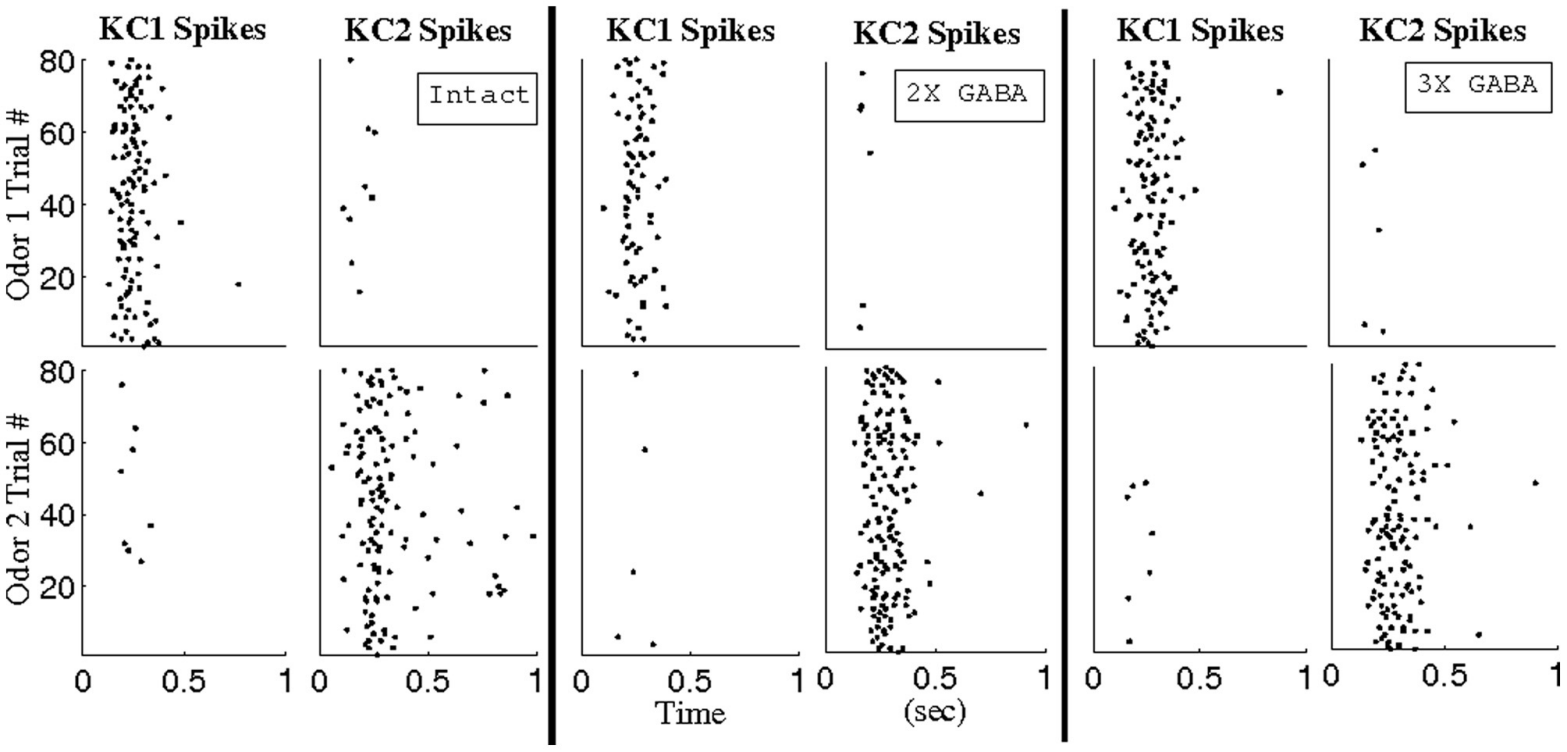
**Reponses of hypothetical Kenyon cells to network activity over 1 s of stimulation.** Analysis was performed in the intact network (I), the intact network with doubled GABA strength (2X GABA), and the intact network with tripled GABA strength (3X GABA). Odors 1 and 2 overlap in 100% of stimulated LNs and 50% of stimulated PNs. Using a BI threshold of ~0.65, PN triplets were extracted from the corresponding networks with no slow inhibition (NS; NS, 2X GABA; NS, 3X GABA) such that a total of 13 PNs comprised the triplets for each odor. KC *k* was designated to have fired whenever 9 of the 13 PNs corresponding to odor *k* spiked within a 10 ms window (*k* = 1, 2).

To test the idea that synchronous PN triplets can be pooled to yield larger subsets of temporally bound neurons, and to ensure that stimulus sensitivity (Figure 7) was not merely an artifact of the arbitrary BI threshold imposed to extract triplets, we constructed hypothetical, odor-specific KCs for a pair of relatively similar odors (100% overlap in the set of stimulated LNs, 50% overlap in the set of stimulated PNs). For each odor, we used a BI threshold of ~0.65 to recover triplets consisting of a total of 13 PNs from networks lacking slow inhibition but with progressively stronger GABA synapses (NS; NS, 2X GABA; NS, 3X GABA), and we designated that the odor *k*-specific KC (KC *k*) fired whenever 9 of the 13 PNs corresponding to odor *k* spiked within a 10 ms window (*k* = 1, 2). Figure 11 plots the spikes of our hypothetical KCs in response to network activity in the presence of functional slow receptors (80 trials per odor); KC *k* responds during every trial of odor *k* but rarely during trials of the other odor. The input–output properties of these hypothetical KCs are in accordance with experimental observations that KCs act as coincidence detectors of presynaptic activity (Perez-Orive et al., 2004). Additionally, responses of our hypothetical KCs generally consist of one or two spikes per second, as observed experimentally in recordings from locust KCs (Perez-Orive et al., 2002).

**Figure 12:**
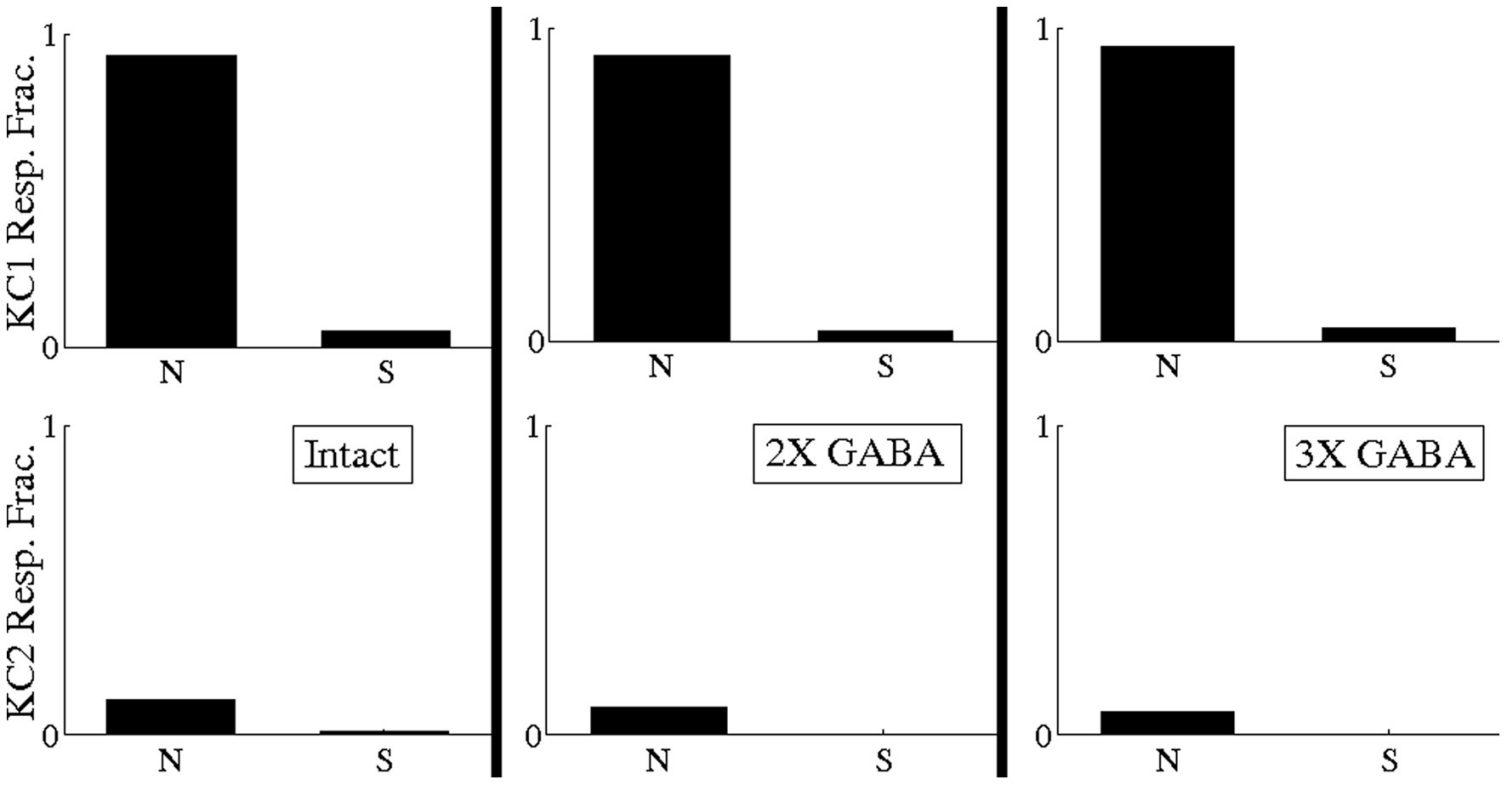
**Fraction of odor 1 trials during which hypothetical Kenyon cells exhibited a response both with (S) and without (N) scrambling of spikes of the 13 temporally bound PNs corresponding to odor 1 (if temporally bound PN *j* spiked *m*_*j*_ times during the first 500 ms of the odor response, we scrambled its spikes by randomly and uniformly redistributing the *m*_*j*_ spikes of PN *j* throughout the first 500 ms of AL network activity).** Analysis was performed in the intact network (I), the intact network with doubled GABA strength (2X GABA), and the intact network with tripled GABA strength (3X GABA). The 13 temporally bound PNs for odor 1 were obtained by imposing a BI threshold of ~0.65 on the corresponding networks with no slow inhibition (NS; NS, 2X GABA; NS, 3X GABA). Analysis was performed over 80 trials.

Though Figure 6 (“Intact” histogram) suggests that temporally bound PNs tend to fire at odor onset with low rate (<20 Hz) in networks with both fast and slow inhibition, it is important to verify that the synchronized PN activity during odor onset driving our hypothetical KCs occurred as a consequence of correlated firing (rather than as a consequence of temporally bound PNs firing with high rate). We therefore measured the responses of our hypothetical KCs to odor 1 after scrambling the spikes of the 13 PNs comprising the temporally bound subset corresponding to odor 1 (i.e., if temporally bound PN *j* spiked *m*_*j*_ times during the first 500 ms of the odor response, we randomly and uniformly redistributed the *m*_*j*_ spikes of PN *j* throughout the first 500 ms of AL network activity). If the responses of KC 1 to odor 1 shown in Figure 11 are a consequence of PN synchrony due to uncorrelated high-rate firing, then scrambling should have little effect on PN synchrony and KC 1 should continue to respond to odor 1 even after scrambling. If, however, the responses of KC 1 to odor 1 shown in Figure 11 are a consequence of PN synchrony due to low-rate correlated firing, then scrambling should eliminate PN synchrony and abolish KC 1 responses. In Figure 12 we plot the fraction of odor 1 trials during which our hypothetical KCs exhibited a response both with (S) and without (N) spike scrambling. Figure 12 shows that KC 1 responds on nearly every trial of odor 1 without scrambling, but responds on only a small fraction of odor 1 trials after spike scrambling, indicating that the PN synchrony driving our hypothetical KCs is due to temporally correlated spiking rather than high firing rates. Thus, we conclude that, within our model, there exist stimulus-specific, temporally bound subsets of ~10 PNs that manifest within a small time window near stimulus onset and are capable of relaying odor information to downstream decoders.

## Discussion

Our results suggest that transient materialization of temporally bound subsets of active neurons may be a device used to encode olfactory information within the AL. The possible existence of such a code, however, naturally leads to several questions that must be addressed. What manner of dynamical interplay within the network contributes to the generation of synchrony? Since temporal binding is transient and triggered on odor onset, how is stimulus information represented once the window of synchronization expires? What are the properties possessed by downstream decoders that enable detection of coherent PN signals? Finally, how viable is a temporal binding code within the AL?

### Dynamics of synchrony

Since GABA synapses lead to odor-evoked, global 20 Hz network oscillations (as seen in the LFP), it follows that LNs within our network exhibit synchronized spiking, with the slow LN calcium spikes giving rise to the 50 ms oscillation time scale. This implies that, under stimulation, PNs receive a periodic inhibitory drive mediated through fast GABA_A_ receptors, with the driving amplitude proportional to the number of active presynaptic LNs. Periodic LN input induces subthreshold membrane potential oscillations in postsynaptic PNs, and the probability of a PN spike is maximized at the peak of the voltage fluctuation. Thus, temporal correlations in spiking activity occur as a consequence of periodic, globally synchronized inhibition; PNs within the network that receive a large-amplitude inhibitory drive (i.e., cells with the largest number of active presynaptic LNs) are strongly constrained to fire within a small neighborhood of peaks in the LFP oscillation. The odor-specific subset of PNs that, in addition to vigorous inhibitory modulation, are also influenced by substantial odor-specific excitatory input, tend to fire at the peak of every oscillation cycle and only at the peak of each oscillation cycle, and hence emerge through network dynamics as a temporally bound neural assembly (see Figure 4; temporally bound PNs fire at ~20 Hz, or once per oscillation cycle). In the presence of a slow inhibitory current, however, the ensemble of coherently spiking neurons is selectively suppressed within ~500 ms of odor onset; this occurs because of the interaction of slow inhibition with slowly rising ORN input. A large number of active LNs are presynaptic to each temporally bound PN, and as ORN input rises, presynaptic LN activity rises as well, and hence slow inhibitory input also rises. Temporally bound PNs, however, continue to spike, since slow inhibition lags behind the rising excitation. Once ORN activity saturates, slow inhibition “catches up” to the excitation and silences temporally bound cells.

The picture presented above of stimulus-sensitive temporal correlations triggered on odor onset and subsequently destroyed via slow dynamics relies implicitly on the identical distribution of fast and slow inhibitory synapses. Since co-localization of GABA receptors with kinetics operating over disparate time scales has been observed in a variety of insect systems (Cayre et al., 1999; Corronc et al., 2002; Barbara et al., 2005; Enell et al., 2007), the assumption that slow and fast receptors have similar distributions within the locust AL seems reasonable.

Theoretical work within the insect AL by Martinez and Montejo (2008) suggests that, when network activity leads to a heterogeneous distribution of both GABA_A_-type and GABA_B_-type inhibitory drive across PNs, fast inhibition exerts a synchronizing influence on postsynaptic cells while slow inhibition tends to have a desynchronizing effect on neuronal spiking. Moreover, the authors show that the extent of synchronization of a particular PN within the network is determined by the ratio of fast to slow inhibitory input amplitude—only neurons that receive substantial fast inhibition and little to no slow inhibition synchronize effectively. Within our AL model, co-localization of GABA_A_-type and GABA_B_-type receptors implies that, after the slow conductance has activated and approached a steady state value, the ratio of fast to slow inhibitory input is constant across PNs. Furthermore, since maximal fast and slow inhibitory conductances are similar within our network, the value of this ratio is ~1, a value which Martinez and Montejo (2008) predict would yield only weak synchrony, as seen in our model. Shortly following stimulus onset (while slow inhibition is relatively weak), however, the ratio of fast to slow inhibitory input magnitude can vary broadly across PNs in an odor-specific manner; cells that receive a large amount of fast inhibition have high ratios and synchronize strongly, while neurons that receive little fast inhibition possess ratios close to the steady-state value of 1 and synchronize weakly. In agreement with the work of Martinez and Montejo (2008), PNs within our network associated with high ratios of fast to slow input emerge as a stimulus-dependent, temporally bound neural assembly (until the slowly-activating inhibitory current reaches peak amplitude).

Previous modeling work by Bazhenov et al. (2001a,b) has examined the role of fast and slow inhibitory time scales within the locust AL. It is important to note that, in contrast to the locust AL model of Bazhenov et al. (2001a,b), our model has sparse connectivity among PNs, weak PN–PN synapses, and a prolonged rise and decay time of ORN input to PNs to match the time course observed experimentally (Wehr and Laurent, 1999). We found that sparse connectivity and weak coupling (or even no coupling) among PNs were required to obtain the uncorrelated ~2–4 Hz spontaneous activity seen in locust PNs (Perez-Orive et al., 2002), while the interplay between the time course of ORN input and slow inhibitory current within our model was essential in generating slow temporal patterning and the principal component trajectories of network activity observed experimentally by Mazor and Laurent (2005); see Patel et al. (2009) for details.

Additionally, while earlier models of Bazhenov et al. (2001a,b) describe short-term correlations (i.e., transient synchrony) in PN activity resulting from fast GABA synapses, our methodology examines the ability of fast GABA synapses to generate long-term correlations in PN activity. In the earlier models of Bazhenov et al. (2001a,b), the set of synchronously firing PNs is updated once every oscillation cycle, but since this change occurs gradually from one cycle to the next, each participating PN will be synchronized to the global oscillation for several consecutive cycles. Two PNs which are synchronized to the global oscillation during overlapping oscillation cycles will therefore exhibit transient correlations in spiking activity—they will exhibit correlated firing during these few overlapping oscillation cycles (spanning 50–150 ms), but not during any other period during the odor response. In contrast, our model asserts that within the evolving population of transiently synchronized PNs there exists a subset of PNs that is permanently synchronized (i.e., a subset of synchronously firing PNs that does not change from one cycle to the next), and that it is this stable “temporally bound” subset that is actually coding for the odor.

Moreover, the models of Bazhenov et al. (2001a,b) suggest that the activation of a slow inhibitory current diminishes synchrony over ~500–1000 ms of the odor response simply by diminishing the total number of network spikes (and hence diminishing the total number of network spikes synchronized to the global oscillation). In contrast, our work asserts that slow inhibition diminishes global synchrony by specifically silencing those PNs which are most strongly phase-locked to the network oscillation; furthermore, we assert that it is precisely these PNs which (while they are active, during the first ~500 ms of odor presentation) form our stable, permanently synchronized (i.e., temporally bound) neural subset. Our work therefore ascribes a specific, physiologically meaningful dynamical role to each inhibitory component: fast GABA input, by tightly synchronizing only a small subset of excited PNs to the network oscillation, determines the composition of the stable temporally bound neural subset that can be used to identify the odor, while slow inhibition selectively suppresses these temporally bound neurons ~500–1000 ms after odor onset, allowing this temporally bound PN subset to be able to specifically signal odor onset.

### Delayed rate coding

During prolonged stimulation, once network oscillations dampen (after 0.5–1 s; Mazor and Laurent, 2005; Patel et al., 2009) and the initial transient synchronous behavior decays, temporally bound neurons are quiescent, and odor-encoding must therefore occur through an alternate mechanism. Accordingly, slow receptor kinetics lead to the emergence of temporally structured PN responses that stabilize 0.5–1 s following stimulus onset, which, when viewed from a rate coding perspective, entail high dimensional odor representations, enhanced stimulus separation, and a broad distribution of PN firing rates (Patel et al., 2009). Thus, during the latter epoch of the odor response, it may be the case that the onset-triggered, transient correlation-based code is superseded by a rate code, with distinct subsets of PNs engaged by the dual coding mechanisms.

While rate-based odor discrimination can allow for highly accurate odor discrimination within our model (Patel et al., 2009), the temporal binding code discussed in the present paper provides several advantages over rate-based odor discrimination. First, temporal binding is more consistent than a rate-based code with the physiology of KCs, which are believed to act as highly precise coincidence detectors of PN activity (see “Downstream Decoders” section). Additionally, rate-based odor discrimination requires hundreds of milliseconds to occur, since rate-based odor discrimination can be effective only once the firing rates of different PNs within the network rise and diverge in an odor-specific manner. Temporal binding, on the other hand, allows for nearly instantaneous odor detection (within the first couple of oscillation cycles), since the temporal binding code can be detected as soon as a synchronous firing event of the odor-specific temporally bound subset occurs, an event which occurs before firing rates within the network change considerably.

It is important to mention that the notion of a rate code is somewhat misleading: rate measurements represent a convenient experimental methodology for studying response intensity, but neural decoders detect individual spikes rather than time-averaged firing rates. It follows that if decoders of PN activity selectively respond to temporally coherent signals, then synchrony is required even during the latter epoch of the AL response to activate downstream elements. Since the network oscillation is dampened, but not abolished, during the latter epoch of the stimulus response, PNs retain a faint tendency toward in-phase spiking (Mazor and Laurent, 2005; Patel et al., 2009). Although individual PNs are likely to display similar weak correlations to the network oscillation, cells with the highest firing rates, simply by virtue of the number of spikes they produce, contribute the majority of phase-locked spikes at every oscillation cycle. In this manner, filters of synchronized input could preferentially detect ensembles of neurons with high firing rates, hence allowing a rate code to be deciphered.

### Downstream decoders

KCs, the neurons of the mushroom body that decode PN activity (Kenyon, 1896; Laurent and Naraghi, 1994), each read from ~400 PNs (Jortner et al., 2007) and display an intrinsic, voltage-dependent non-linearity that selectively amplifies coincident input (Perez-Orive et al., 2004). Odor-evoked barrages of globally synchronized PN spikes impinge upon both KCs and LHIs, which are GABAergic interneurons located in a structure called the lateral horn (Hansson and Anton, 2000). Since KC dendrites are known to receive GABAergic input (Leitch and Laurent, 1996), and LHI axon collaterals have been shown to diffusely overlap KC dendrites, LHIs were thought to be the source of the strong, periodic, phase-delayed inhibition seen in recordings from KCs (Perez-Orive et al., 2002), though recent experiments suggest that a giant GABAergic interneuron (GGN) within the mushroom body, rather than LHIs, provides the inhibition seen in KC recordings (Papadopoulou et al., 2011; Gupta and Stopfer, 2012). Thus, KCs receive coherent PN input in 50 ms epochs, and toward the end of each epoch the membrane potential of every KC is effectively reset by incoming inhibition. The properties of these downstream elements imply that they filter coincident spikes through a small time window occurring early in each cycle of network oscillation, and that there is minimal interaction between PN signals in successive cycles.

In light of the physiology of the neurons that decode PN output, our model makes several predictions about KC responses that can be evaluated in relation to available data. Consistent with experiment (Perez-Orive et al., 2002), our hypothetical, odor-specific KCs respond sparsely to their preferred stimuli, with only a few action potentials elicited during 1 s of stimulation. Furthermore, since binding-induced PN coherence emerges from network dynamics within a fleeting, onset-triggered time window, after which a rate code involving a disparate set of PNs ensues (which is less efficient at driving coincidence detectors), our model predicts that the intensity of the KC population response is maximal within ~500 ms of odor onset, and that distinct subsets of KCs fire initially and after extended odor exposure. Recordings from mushroom body cells verify this prediction; locust KCs are most active transiently following stimulus onset, and the distribution of response latencies suggests that individual KCs show a preference for either early or late phases of odor-evoked AL activity (Mazor and Laurent, 2005, Figure 7E; Perez-Orive et al., 2004, Figure 5C).

### Temporal binding as an odor code

The existence of a neural code in which a salient sensory feature is represented by temporally binding together the spikes of a precise subset of active neurons (without altering the firing rates of individual neurons within the network) was first postulated by von der Malsburg (1981). von der Malsburg (1981) pointed out that the advantage of such a code is the ability to simultaneously signal both the particular sensory stimulus being represented (by the temporal binding of spikes pertaining to that stimulus) as well as various stimulus attributes (by modulating the firing rates of individual cells taking part in the temporally bound neural assembly). Since its inception, the notion of temporal binding as a neural coding tool has received a great deal of attention, from being invoked as a mechanism used by the visual cortex for feature binding (Eckhorn et al., 1988; Gray et al., 1989; Singer, 1993; Singer and Gray, 1995; Engel et al., 1997; Roelfsema and Singer, 1998; Herculano-Houzel et al., 1999) to being posited as the neural correlate of sensory attention (Niebur et al., 1993).

In the much simpler context of the locust AL, temporal binding may play a role in the encoding of stimulus features such as odor identity or concentration. Furthermore, the primary obstacles to the utilization of a temporal binding code—the combinatorial explosion associated with the required number of read-out cells (or cardinal neurons) and the potential confound posed by synchronized spiking events that occur due to chance rather than correlated firing (Shadlen and Movshon, 1999)—are circumvented by the functional design of the locust olfactory system. There are a large number of KCs (~50,000) in the locust mushroom body that read from the 830 PNs in a combinatorial manner (Jortner et al., 2007) and are thought to act as coincidence detectors of synchronized PN input (Perez-Orive et al., 2004), which puts these cells in an ideal position to act as the cardinal neurons needed to decode temporally coherent signals. Additionally, stimulus-evoked firing rates in the AL are relatively low, on the order of ~20 Hz (Perez-Orive et al., 2002), implying that a synchronized spiking event involving several cells is unlikely to occur in the absence of correlated PN activity.

Within the context of the locust AL, our proposed temporal binding strategy allows for rapid and fine odor discrimination (based on KC firing events within one or two oscillation cycles), as well as the ability to differentiate between the appearance of a novel odor versus sustained exposure to an odor within the environment (through the presence/absence of specific KC activation patterns). Stopfer et al. (2003) recorded from multiple PNs within the locust AL and were unable to find evidence of a stable, odor-specific subset of active PNs. However, Stopfer et al. (2003) looked for correlated PN spiking using 50 ms time windows. In order to detect the correlated activity that we describe in our work, one would have to use smaller time windows (10–20 ms windows rather than the full oscillation cycle length of 50 ms). In fact, if we look for correlated activity within our model using 50 ms windows then we find no correlations (i.e., we do not find temporal binding). Correlated activity is found within our model only when we look for correlations over small enough time windows of 10–20 ms. Furthermore, Stopfer et al. (2003) pool data from multiple animals, a practice which is likely a valid technique when one resolves PN activity using 50 ms time windows; however, pooling data from multiple animals would make it difficult to detect correlated activity that occurs over the shorter time scales that our temporal binding hypothesis requires. Thus, we propose that the temporal evolution of synchronized PN ensembles that emerges through network dynamics actually masks an underlying binding code. To directly observe a temporally bound PN subset, simultaneous recordings from a large number of PNs (~100) within a single animal would need to be performed, an experiment which may be technically difficult. Indirect tests of the temporal binding hypothesis, however, may be performed—e.g., via simultaneous recordings from pairs of PNs. If a PN pair were part of a temporally bound subset, then the two PNs would exhibit long-term correlations in precise spike timing followed by quiescence during the latter portion of the odor response. Recordings from a sufficiently large number of PN pairs would be highly likely to encounter a temporally bound pair, if a temporally bound neural subset exists.

## Methods

The model network consisted of 90 PNs and 30 LNs, in accordance with the experimentally observed ratio of approximately three PNs to one LN in the locust AL (Leitch and Laurent, 1996). The membrane potential of each PN and LN was governed by a single-compartment equation obeying Hodgkin–Huxley type kinetics. The PN and LN currents were taken from those used by Bazhenov et al. (2001a,b) in their locust AL model.

### Network properties

The network consisted of randomly interconnected PNs and LNs with cell-type specific connection probabilities. The PN–PN and PN–LN connection probability was 0.1, while the LN–LN connection probability was 0.25 and the LN–PN connection probability was 0.15. Although sparse PN–PN connections were required for consistency with experiment, similar network dynamics could be obtained when all other connections were considerably denser, as described in our previous paper (Patel et al., 2009). Each PN received background current input in the form of a Poisson spike train with a mean rate of 3500 spikes/s and a spike strength of 0.0654 μA. In agreement with experiment, this resulted in a background PN firing rate of approximately 2–4 spikes/s (Perez-Orive et al., 2002). All simulations were performed using the explicit Euler method with a time step of 0.01 ms.

### Currents and equations

Each PN was equipped with Hodgkin–Huxley sodium and potassium spiking currents as well as a transient potassium current. LNs in the locust AL, however, do not generate traditional action potentials; rather, LNs exhibit slow 20–30 ms calcium spikes that decrease in frequency after 100–200 ms of steady stimulation (Laurent et al., 1993). Thus, LNs in our model network were equipped with a slow calcium current to reproduce the 20–30 ms spikes, a calcium-dependent potassium current to allow for spike adaptation, and a traditional potassium current.

PN cholinergic synapses and LN GABAergic synapses were modeled by fast-activating synaptic currents. While cholinergic transmission was modeled via stereotyped neurotransmitter release in response to a presynaptic PN action potential, a continuous coupling model was used to simulate GABAergic transmission—neurotransmitter release was dependent upon the level of presynaptic LN depolarization (Laurent et al., 1993). Additionally, a slow inhibitory synaptic current from LNs to PNs was introduced in order to reproduce the slow temporal patterns observed experimentally in PN odor responses (Laurent et al., 1996). The current was modeled as acting through slowly-activating inhibitory receptors and required a series of approximately three LN calcium spikes to become active.

The membrane potential of each PN and each LN is governed by equations of the following form:

$$\begin{array}{l} C_{m}\frac{dV_{\text{PN}}}{dt}=-g_{L}(V_{\text{PN}}-E_{L})-I_{\text{Na}}-I_{\text{K}}-I_{\text{A}}\\ \;-I_{\text{GABA}}-I_{\text{slow}}-I_{\text{nACH}}-I_{\text{stim}}\\ C_{m}\frac{dV_{\text{LN}}}{dt}=-g_{L}(V_{\text{LN}}-E_{L})-I_{\text{Ca}}-I_{\text{CaK}}-I_{\text{K}}\\ \;-I_{\text{GABA}}-I_{\text{nACH}}-I_{\text{stim}}. \end{array}$$

The parameters for the passive leak current were *C*_*m*_ = 1.0 μF/cm^2^, *g*_*L*_ = 0.3 mS/cm^2^, *E*_*L*_ = −64 mV for PNs and *C*_*m*_ = 1.0μF/cm^2^, *g*_*L*_ = 0.3 mS/cm^2^, *E*_*L*_ = − 50 mV for LNs.

#### Intrinsic currents

The intrinsic currents consisted of fast sodium and potassium currents *I*_Na_ and *I*_K_, a transient calcium current *I*_Ca_, a calcium-dependent potassium current *I*_CaK_, and a transient potassium current *I*_A_. All such currents obeyed equations of the following form:

$$\begin{array}{l} I_{j}=g_{j}m^{M_{j}}h^{N_{j}}(V-E_{j})\\ \;j\in \{\text{Na},\text{K},\text{Ca},\text{CaK},\text{A}\}. \end{array}$$

The maximal conductances were *g*_Na_ = 120 mS/cm^2^, *g*_K_ = 3.6 mS/cm^2^, *g*_A_ = 1.43 mS/cm^2^ for PNs and *g*_Ca_ = 5.0 mS/cm^2^, *g*_CaK_ = 0.045 mS/cm^2^, *g*_K_ = 36 mS/cm^2^ for LNs. The reversal potentials were *E*_Na_ = 40 mV, *E*_K_ = −87 mV for PNs and *E*_Ca_ = 140 mV, *E*_K_ = −95 mV for LNs.

The gating variables *m*(*t*) and *h*(*t*) take values between 0 and 1 and obey the following equations:

$$\begin{array}{l} \frac{dm}{dt}=\frac{m_{\infty }(V)-m}{\tau_{m}(V)}\\ \frac{dh}{dt}=\frac{h_{\infty }(V)-h}{\tau_{h}(V)}. \end{array}$$

*I*_Na_ and *I*_K_ are described in Hodgkin and Huxley (1952).

The *I*_Ca_ current has *M*_Ca_ = 2, *N*_Ca_ = 1, *m*_∞_ = 1/(1 + exp(−(*V* + 20)/6.5)), τ_*m*_ = 1 + (*V* + 30)0.014 ms, *h*_∞_ = 1/(1 + exp((*V* + 25)/12)), τ_*h*_ = 0.3 exp((*V* − 40)/13) + 0.002 exp(−(*V* − 60)/29) ms (Laurent et al., 1993).

The *I*_CaK_ current has *M*_CaK_ = 1, *N*_CaK_ = 0, *m*_∞_ = [Ca]/([Ca] + 2), τ_*m*_ = 100/([Ca] + 2) ms (Sloper and Powell, 1978).

The *I*_A_ current has *M*_A_ = 4, *N*_A_ = 1, *m*_∞_ = 1/(1 + exp(−(*V* + 60)/8.5)), τ_*m*_ = (0.27/(exp((*V* + 35.8)/19.7) + exp(−(*V* + 79.7)/12.7)) + 0.1) ms, *h*_∞_ = 1/(1 + exp((*V* + 78)/ 6)), τ_*h*_ = 0.27/(exp((*V* + 46)/5) + exp(−(*V* + 238)/37.5)) ms for *V* < −63 mV and τ_*h*_ = 5.1 ms for *V* > −63 mV (Huguenard et al., 1991).

The dynamics of intracellular calcium concentration [Ca] were governed by the following equation:

$$\frac{d[\text{Ca}]}{dt}=-AI_{T}-\frac{[\text{Ca}]-{\left[\text{Ca}\right]}_{\infty }}{\tau },$$

where [Ca]_∞_ = 0.00024 mM, *A* = 0.0002 mM × cm^2^/(ms × μA), and τ = 150 ms.

#### Synaptic currents

The GABA currents for a neuron *p* were governed by equations of the following form:

$$\begin{array}{l} I_{\text{GABA}}=g_{\text{GABA}}[O](V-E_{\text{GABA}})\\ \;[O]=\sum_{q\in Y}{\left[O\right]}_{q} \end{array}$$

where *Y* = {set of all LNs that synapse onto *p*}. For a synapse from LN *q* onto neuron *p*, the fraction of open channels [*O*]_*q*_ obeyed the equation

$$\frac{d{\left[O\right]}_{q}}{dt}=\alpha (1-{\left[O\right]}_{q}){\left[T\right]}_{q}-\beta {\left[O\right]}_{q}.$$

Since LNs exhibit slow calcium potentials rather than traditional all-or-none spikes and GABA release is a continuous function of presynaptic membrane potential, neurotransmitter release by LN *q* ([*T*]_*q*_) was governed by the equation

$${\left[T\right]}_{q}=\frac{1}{1+\text{exp}(-(V_{q}(t)-V_{0})/\sigma )}.$$

The reversal potential was *E*_GABA_ = −70 mV and the rate constants were α = 10 ms^−1^ and β = 0.16 ms^−1^. The parameters for [*T*]_*q*_ were *V*_0_ = −20 mV and σ = 1.5 (Bazhenov et al., 2001b).

The nicotinic acetylcholine currents for a neuron *p* were governed by equations of the following form:

$$\begin{array}{l} I_{\text{nACH}}=g_{\text{nACH}}[O](V-E_{\text{nACH}}).\\ \;[O]=\sum_{q\;\in Y}{\left[O\right]}_{q} \end{array}$$

where *Y* = {set of all PNs that synapse onto *p*}. For a synapse from PN *q* onto neuron *p*, the fraction of open channels [*O*]_*q*_ obeyed the equation

$$\frac{d{\left[O\right]}_{q}}{dt}=\alpha (1-{\left[O\right]}_{q}){\left[T\right]}_{q}-\beta {\left[O\right]}_{q}.$$

Neurotransmitter release by PN *q* ([*T*]_*q*_) was governed by the equation

$${\left[T\right]}_{q}=0.5\theta (t_{0}+t_{\text{max}}-t)\theta (t-t_{0}).$$

The reversal potential was *E*_nACH_ = 0 mV and the rate constants were α = 10 ms^−1^ and β = 0.2 ms^−1^. θ(*x*) is the Heaviside step function, *t*_0_ is the time of receptor activation, *t*_max_ = 0.3 ms, *V*_0_ = −20 mV, and σ = 1.5. Receptor activation was determined to have occurred when the membrane potential of PN *q* crossed a threshold of zero mV from below (Bazhenov et al., 2001b).

The slow inhibitory currents for a PN *p* were governed by the following scheme:

$$\begin{array}{l} I_{\text{slow}}=g_{\text{slow}}\frac{{\left[G\right]}^{4}}{{\left[G\right]}^{4}+K}(V-E_{\text{K}})\\ {}[G]=\sum_{q\in Y}{\left[G\right]}_{q} \end{array}$$

where *Y* = {set of all LNs that synapse onto *p*}. For a synapse from LN *q* onto neuron *p*, the fraction of activated receptors [*R*]_*q*_ and the concentration of receptor coupled G proteins [*G*]_*q*_ were governed by the equations

$$\begin{array}{l} \frac{d{\left[G\right]}_{q}}{dt}=r_{3}{\left[R\right]}_{q}-r_{4}{\left[G\right]}_{q}.\\ \frac{d{\left[R\right]}_{q}}{dt}=r_{1}(1-{\left[R\right]}_{q}){\left[T\right]}_{q}-r_{2}{\left[R\right]}_{q}\\ \;{\left[T\right]}_{q}=0.5\theta (t_{0}+t_{\text{max}}-t)\theta (t-t_{0}). \end{array}$$

The reversal potential was *E*_K_ = −95 mV and the rate constants were *r*_1_ = 0.5 mM^−1^ms^−1^, *r*_2_ = 0.0013 ms^−1^, *r*_3_ = 0.1 ms^−1^, *r*_4_ = 0.033 ms^−1^, and *K* = 100 μM^4^. θ(*x*) is the Heaviside step function, *t*_0_ is the time of receptor activation, and *t*_max_ = 0.3 ms. Receptor activation was determined to have occurred when the membrane potential of LN *q* crossed a threshold of zero mV from below (Destexhe et al., 1996; Bazhenov et al., 1998).

Maximal synaptic conductances were *g*_GABA_ = 0.3 mS/cm^2^ from LNs to LNs, *g*_GABA_ = 0.36 mS/cm^2^ and *g*_slow_ = 0.36 mS/cm^2^ from LNs to PNs, *g*_nACH_ = 0.045 mS/cm^2^ from PNs to LNs, and *g*_nACH_ = 0.009 mS/cm^2^ from PNs to PNs.

### Odor simulation

An odor was simulated by stimulating a set of 36 PNs and 12 LNs. Each stimulated cell received stimulus current (via the *I*_stim_ term above) in the form of 200 independent Poisson spike trains, each with a mean rate of 35 spikes/s and a spike strength of 0.01743 μA (PNs) or 0.01667 μA (LNs). Due to the large convergence ratio of ORN inputs onto PNs in the locust (Homberg et al., 1989; Hildebrand et al., 1997; Mazor and Laurent, 2005) and their mean-driven log-linear response properties (Rubin and Katz, 1999; Duchamp-Viret et al., 2000; Meister and Bonhoeffer, 2001; Wachowiak and Cohen, 2001; Reisenman et al., 2004; Hallem and Carlson, 2006), we modeled ORN input to each AL neuron as a stochastic process (with Poisson statistics) rather than simulating individual ORNs explicitly. Consistent with experiment, PNs which were active during stimulus presentation exhibited firing rates of 10–40 spikes/s (Perez-Orive et al., 2002). Eighty trials were performed for each stimulus with a 10 s total duration for each trial. Stimulus onset occurred at *t*_*o*_ = 1 s and stimulus offset occurred at *t*_*d*_ = 3.5 s. In order to capture the experimentally observed time course of ORN input to the locust AL (Wehr and Laurent, 1999), we modeled stimulus rise as exponential with a rise time of 400 ms, while stimulus decay was modeled as root exponential with a decay time of approximately 1000 ms. The odor-evoked input rate of ORN spikes to a stimulated cell in the network was given by *R*(*t*) = *r*_*m*_ exp(−(*t* −(*t*_*o*_ + *s*))^2^/*c*_1_) for *t* = *t*_*o*_ to *t* = *t*_*o*_ + *s*, by *R*(*t*) = *r*_*m*_ for *t* = *t*_*o*_ + *s* to *t* = *t*_*d*_, and *R*(*t*) = *r*_*m*_ exp(−sqrt(*t* − *t*_*d*_)/*c*_2_) for *t* > *t*_*d*_, where *s* = 400 ms was the rise time, *c*_1_ = 100,000, *c*_2_ = sqrt(1000) were the scaling constants, and *r*_*m*_ was the maximal stimulus-evoked ORN input rate (described above).

It is generally thought that the olfactory system initially encodes odors in a combinatorial manner—different odors are represented by differing (but potentially overlapping) subsets of active ORNs (Joerges et al., 1997; Vickers and Christensen, 1998; Vickers et al., 1998; Malnic et al., 1999; Ng et al., 2002; Wang et al., 2003; Ache and Young, 2005). We therefore simulated different odors by stimulating varying subsets of 36 PNs and 12 LNs, with the statistics of current input (described above) uniform across stimulated cells.

### Synchrony ratio

In order to assess whether a triplet of PNs exhibited correlated firing (i.e., fired together more often than would be expected from the individual PN spike rates) we devised a measure that we termed the SR. For an ordered triplet *i*,*j*,*k* of PNs, we defined the conditional probability *P*_*j*, *k*|*i*_ as the probability that *j* and *k* fire given that *i* fires, and similarly we defined *P*_*j*|*i*_ and *P*_*k*|*i*_ as the probability that *j* fires given that *i* fires and the probability that *k* fires given that *i* fires, respectively. To compute *P*_*j*, *k*|*i*_, we looked in a 20 ms window centered at every spike time of *i* during the first second of the network response after stimulus onset in all 80 trials for the given stimulus. If both *j* and *k* fired within one of these 20 ms windows, we designated that a synchronized firing event had occurred, and *P*_*j*, *k*|*i*_ was computed as the number of synchronized firing events divided by the total number of times that *i* spiked. *P*_*j*|*i*_ was computed in a similar fashion, except that a synchronized firing event was determined to have occurred every time that *j* fired within a 20 ms window centered at a spike time of *i* (*P*_*k*|*i*_ was computed in an analogous manner). The SR of the ordered triplet *i*,*j*,*k* was then determined by the formula $SR_{j,k|i}=\frac{P_{j,k|i}}{P_{j|i}P_{k|i}}-1$.

Because we were ultimately interested in temporally bound PN subsets, we only considered ordered triplets *i*,*j*,*k* with *P*_*j*|*i*_ > 0.5 and *P*_*k*|*i*_ > 0.5. Since by construction *P*_*j*, *k*|*i*_ cannot exceed min(*P*_*j*|*i*_, *P*_*k*|*i*_), the SR must satisfy −1 < SR_*j*, *k*|*i*_ < 1. In the case that, conditioned on spikes of *i*, PNs *j* and *k* fire independently of each other, we would expect that *P*_*j*, *k*|*i*_ = *P*_*j*|*i*_ *P*_*k*|*i*_, and so *SR*_*j*, *k*|*i*_ should be close to zero. If, however, PNs *j* and *k* exhibit correlated conditional firing, then we would expect *P*_*j*, *k*|*i*_ > *P*_*j*|*i*_ *P*_*k*|*i*_, with *P*_*j*, *k*|*i*_ approaching the value min(*P*_*j*|*i*_, *P*_*k*|*i*_) as the degree of correlation becomes stronger. Thus, the deviation of the synchrony ratio *SR*_*j*, *k*|*i*_ from zero provides a measure of correlated firing among PNs *i*,*j*,*k*, with values approaching −1 implying negative correlations and values approaching +1 indicating positive correlations.

### Binding index

In an effort to quantify temporal binding in a more direct fashion, we constructed a measure that we termed the binding index. For a triplet *i*,*j*,*k* of PNs (independent of ordering), we defined the binding index by the formula BI_*i*, *j*, *k*_ = min(*P*_*j*, *k*|*i*_, *P*_*i*, *j*|*k*_, *P*_*i*, *k*|*j*_). It therefore follows that if the triplet *i*,*j*,*k* is described by a binding index of BI_*i*, *j*, *k*_ = *b*, then whenever either one of the PNs *i*, *j*, or *k* fires the other two PNs will fire concurrently with at least a probability *b*. We also computed a binding index for PN quadruplets in a similar manner, i.e., BI_*i*, *j*, *k*, *m*_ = min(*P*_*j*, *k*, *m*|*i*_, *P*_*i*, *j*, *m*|*k*_, *P*_*i*, *k*, *m*|*j*_, *P*_*i*, *j*, *k*|*m*_), where the conditional probabilities involving four PNs in the formula represent obvious extensions of the definitions given above.

### Symmetric difference ratio

We designed the SDR to measure the degree of similarity between two subsets *A* and *B* of PNs, a normalized quantity where values approaching 1 imply that the sets *A* and *B* are nearly disjoint and values approaching 0 imply that *A* and *B* are nearly identical. If the subset *A* contains *n* PNs, the subset *B* contains *k* < = *n* PNs, and the subsets *A* and *B* together contain a total of *r* distinct PNs with *s* PNs present in both subsets, then the SDR is given by the formula $\text{SDR}=\frac{r-s}{n+k}-\frac{n-k}{n+k}=\frac{2(k-s)}{n+k}$. The second term in the formula removes the contribution to the SDR of the difference in cardinality of the subsets *A* and *B*.

### Conflict of interest statement

The authors declare that the research was conducted in the absence of any commercial or financial relationships that could be construed as a potential conflict of interest.
